# Molecular determinants of STEC-HUS: from complement activation to microvascular thrombosis

**DOI:** 10.3389/fimmu.2026.1749811

**Published:** 2026-03-13

**Authors:** Donata Santarsiero, Miriam Galbusera, Sara Gastoldi, Elena Bresin, Rossella Piras, Marta Alberti, Marina Vivarelli, Silvia Prandini, Sara Conti, Federica Zotta, Anna Schubart, Ariela Benigni, Giuseppe Remuzzi, Marina Noris, Sistiana Aiello

**Affiliations:** 1 Istituto di Ricerche Farmacologiche Mario Negri IRCCS, Clinical Research Center for Rare Diseases Aldo e Cele Daccò and Centro Anna Maria Astori, Science and Technology Park Kilometro Rosso, Bergamo, Italy; 2 Division of Nephrology and Dialysis, Bambino Gesù Children’s Hospital IRCCS, Rome, Italy; 3 Department of Immunology, Novartis BioMedical Research, Basel, Switzerland

**Keywords:** alternative pathway, complement system, endothelial cells, STEC-HUS, thrombus formation

## Abstract

Shiga-like toxin-producing E. coli-induced hemolytic uremic syndrome (STEC-HUS) is a rare but severe disease characterized by microangiopathic hemolysis, thrombocytopenia, and renal failure. No specific therapy is available, and long-term complications are common. Growing evidence indicates that STEC-HUS is associated with excessive complement activation, however the impact on disease pathogenesis is still debated. This study investigated the involvement of the three complement pathways in STEC-HUS. We analyzed 37 patients during the acute phase and 24 patients after hospital discharge. *Ex-vivo* assays with patient sera and cultured microvascular endothelial cells demonstrated that acute-phase sera triggered abnormal C3 and C5b-9 deposition, leading to increased cell surface expression of vWF and P-selectin, which in turn promoted thrombus formation on endothelial cells. The factor B inhibitor iptacopan, but not inhibitors of classical or lectin pathways, effectively blocked complement deposition and prevented thrombus formation, highlighting the alternative pathway as a driver of complement dysregulation and microvascular thrombosis in acute STEC-HUS. Additionally, we observed persistent complement activation in a substantial subset of patients studied after hospital discharge, as indicated by abnormal C5b-9 formation. Notably, most of these patients had not achieved full remission, showing at least one hematologic abnormality and/or elevated serum creatinine. These findings emphasize the pivotal role of complement overactivation in STEC-HUS pathogenesis and support the potential of alternative pathway inhibitors as promising therapeutic options. Moreover, our results underscore the potential of the *ex-vivo* tests as valuable tools for monitoring complement activity and clotting abnormalities over time, possibly facilitating detection and management of disease sequelae.

## Introduction

The term hemolytic uremic syndrome (HUS) defines a heterogeneous group of rare but severe diseases primarily affecting the kidneys (1). HUS is clinically characterized by hemolytic anemia, thrombocytopenia and end-organ ischemia, due to thrombosis in small blood vessels, such as the glomerular capillaries (2). HUS is most commonly triggered by infections by Shiga-toxin (Stx) producing *Escherichia coli* strains (STEC) and is often preceded by bloody diarrhea. The overall incidence of STEC-HUS is around 2 cases per 100,000 people with higher rates in children less than 5-year-old (3). STEC-HUS is the leading cause of acute kidney injury in children, though severe cases have also been reported in adults (4). Around 70% of the cases recover completely from the acute episode, however the fatality rate is still around 1-5% (5) and 20 to 30% of patients experience varying degrees of long-term sequelae, including hypertension, neurological symptoms and chronic kidney disease, which often necessitate life-long medical and/or rehabilitative care (6–10).

The development of microthrombi in HUS is secondary to endothelial damage which, in the case of STEC-HUS, is caused primarily by Stx. STEC bacteria bind to intestinal epithelial cells and release Stxs that are translocated into blood where they circulate bound to blood cells and their microvesicles. Stx binds glomerular endothelial cells and generates a cascade of signals resulting in inflammation, cell damage and loss of endothelial thromboresistance (11–13).

Pathways activated by Stx include the complement system, a cascade of circulating and cell-surface molecules involved in the immediate response against foreign and altered host cells (14). This possibility has been suggested by the detection of increased circulating levels of complement activation products (15, 16) and of complement-coated microvesicles (17) in STEC-HUS patients during the acute phase.

A specific therapy for STEC-HUS is still lacking and management of this disease is supportive, including control of electrolytes, water balance, hypertension, anemia and renal failure, often requiring dialysis (18). The evidence that complement activation may have a pathogenic role in STEC-HUS has led to the consideration of eculizumab - an anti-C5 monoclonal antibody that effectively induces disease remission in the genetic form of HUS (atypical HUS) - for treating severe cases (19–23). However, nowadays, the use of eculizumab in STEC-HUS is debated, and the optimal management of STEC-HUS remains unresolved (24–27).

In this study, we analyzed the activation of complement cascade and its role in inducing microvascular thrombosis in a cohort of patients diagnosed with STEC-HUS, aiming to deepen the understanding of both the terminal complement pathway and the early steps of complement activation in STEC-HUS pathogenesis, and provide insights that could guide the development of new therapeutic strategies.

## Methods

### Study participants

Participants were patients with diagnosis of STEC-HUS included in the International Registry of Recurrent and Familial Hemolytic Uremic Syndrome/Thrombotic Thrombocytopenic Purpura (HUS/TTP), who were analyzed in our laboratories between 2013 and 2023. The Registry was established in 1996 at the Aldo e Cele Daccò Clinical Research Center for Rare Diseases (Ranica, Bergamo) (villacamozzi.marionegri.it/seu). We also analyzed some patients for whom we already have samples collected and stored at the Mario Negri Institute Biological Resources Center, in Biobank for Rare Diseases and Kidney Diseases (Ranica, Bergamo) and who fulfilled the inclusion criteria. Written informed consent was obtained from all enrolled patients or their guardians.

STEC-HUS patients were evaluated during the acute episode, before receiving plasma infusion/exchange or eculizumab treatment (n = 37), or after hospital discharge, following resolution of infection and at least 8 weeks without treatment prior to the time of serum collection (follow-up, n = 24). Among these, 11 patients were studied at both time points. **Supplementary Figure 1** shows a flow chart summarizing the number of STEC-HUS patients analyzed at each step of the study.

STEC-HUS was diagnosed in all cases reported to have one episode of microangiopathic hemolytic anemia and thrombocytopenia defined on the basis of hematocrit (Ht) <30%, hemoglobin (Hb) <10g/dL, serum lactate dehydrogenase (LDH) >500UI/L, undetectable haptoglobin (Hp), fragmented erythrocytes in the peripheral blood smear, and platelet count <150,000/µL, associated with acute renal failure (serum creatinine >1.3 mg/dL for adults, >0.5 mg/dL for children below 5 years of age and >0.8 mg/dL for children aged 5-10, and/or urinary protein/creatinine ratio >200 mg/g; or an increase serum creatinine or urinary protein/creatinine ratio >15% compared to baseline levels). Evidence of Enterohemorrhagic *E. coli* infection by O157:H7 *E. coli* or any other Stx producing *E. coli* serotype was documented by one of more of the following: presence of Stx-*E. coli* by colorless sorbitol-MacConkey stool cultures, Stx detection in stools by Vero cell assay, presence of *stx* and *eae* genes identified by PCR, detection of circulating anti-*E. coli* (anti-Stx or anti-LPS O157, O26, O111, O145 or any other Stx producing *E. coli* serotype) antibodies by ELISA. The Stx-*E. coli* tests were processed centrally by the Food Safety, Nutrition and Veterinary Public Health Department, Istituto Superiore di Sanità, Rome, Italy.

Thrombotic thrombocytopenic purpura was ruled on the basis of ADAMTS13 activity >10% and no anti-ADAMTS13 antibodies.

Patients studied after hospital discharge and free from STEC infection were defined as patients in full or partial remission. Full remission was defined as normalization of both hematological parameters (Hp between 49 and 246 mg/dL; Hb >10 g/dL; LDH <500 UI/L; platelets >150,000/μL) and renal function (serum creatine 0.3-0.5 mg/dL for children <1–5 years; 0.5-0.8 mg/dL for children aged 5 to 10 years; 0.5-1.2 mg/dL for children >10 years and adults); partial remission was defined as absence of normalization (on the basis of age) of at least one hematological parameter (Hp, Hb, LDH or platelets) or normalization of hematological parameters but with residual renal dysfunction (serum creatinine out of age-based normal ranges).

For 41 patients, genetic testing was available. **Supplementary Table 1** summarizes genetic characteristics of the patients. Rare variants or the homozygous CFHR3/CFHR1 deletion were identified in 6/41 (15%). The screening of coding sequences of aHUS-associated genes (CFH, CFHR1, MCP, CFI, CFB, C3, THBD and DGKE) and candidate genes (CFHR2, CFHR3, CFHR4, CFHR5, and C5) was performed using amplicon-based next-generation sequencing (28). Rare functional variants (missense, nonsense, indel, or splicing variants) with minor allele frequency (MAF) <0.001 in the Genome Aggregation Database (gnomAD, https://gnomad.broadinstitute.org/) were selected. Stop-gain, frameshift and splicing variants, and missense variants with published functional studies, were categorized as pathogenic variants (PV). The other variants were categorized as likely pathogenic variants (LPV), variants of uncertain significance (VUS), likely benign or benign, using guidelines from the American College of Medical Genetics and Genomics (ACMG) and from the KDIGO conference on aHUS and C3G (29–31).

Serum samples from four patients with diagnosis of Systemic Lupus Erythematosus (SLE) and three patients with acute atypical HUS (aHUS) were also included as positive control for specific assays. Sera from 10 different healthy subjects were pooled to prepare the normal human serum pool (NHS). NHS was run in each experiment as a reference (100%) for the stainings. Among the healthy subjects, some volunteers also donated blood used for thrombus formation experiments.

The study was approved by the Comitato Etico di Bergamo ASST-Papa Giovanni XXIII, Bergamo, Italy.

### *Ex vivo* studies with human microvascular endothelial cell line

Human microvascular endothelial cell line of dermal origin (HMEC-1, ATCC CRL-3243) was cultured as described (32). HMEC-1 were plated on glass slides and used when confluent. The cellular morphology, confluence and state were assessed with phase contrast microscopy.

Before each experiment, the cells were incubated overnight with serum free MCDB 131 culture medium supplemented with 10 µg/mL hydrocortisone (Sigma Aldrich), 100 U/mL penicillin, 100 µg/mL streptomycin, 2 mM glutamine (Life Technologies Italia), and 50 µg/mL endothelial cell growth supplement (ECGS) from bovine neural tissue (Sigma Aldrich).

For all the experiments, cells were left unstimulated or activated with 10 µM ADP (Sigma Aldrich) for 10 minutes and then incubated at 37°C for 4 hours with serum (from patients or controls) diluted 1:2 with test medium (Hank’s balanced salt solution, HBSS: 137 mM NaCl, 5.4 mM KCl, 0.7 mM Na_2_HPO_4_, 0.73 mM KH_2_PO_4_, 1.9 mM CaCl_2_, 0.8 mM MgSO_4_, 28 mM Trizma base pH 7.3, 0.1% dextrose; with 0.5% bovine serum albumin BSA). In selected slides of specific experiments, the mix of serum and test medium was added with: soluble complement receptor 1 (sCR1, also known as CD35, 150 µg/mL, R&D Systems), a pan-complement inhibitor (33, 34); or an anti-MASP2 antibody (2 µg/mL, Invitrogen), an inhibitor of the LP; or C1-inhibitor (36 µg/mL, Sigma Aldrich) (35), a recombinant serine protease inhibitor that mainly inhibits the CP; or an inhibitor of factor B (1 µM or 10 µM, iptacopan, LNP023, Novartis) (36) that selectively inhibits the AP; or eculizumab (100 µg/mL, Soliris^®^, Alexion) that blocks the cleavage of C5.

In each experiment, a pool of sera from 10 healthy controls (normal human serum, NHS) was run in parallel as a reference (100%) for stainings.

At the end of the incubation step, HMEC-1 were washed twice and then utilized for immunofluorescence studies (staining of C3, C5b-9, C4, IgG, P-selectin or von Willebrand factor) or experiments of thrombus formation.

### Immunofluorescence studies

At the end of the incubation step, HMEC-1 were washed twice and fixed in 3% paraformaldehyde. After 2 washes, blocking was carried out with 2% bovine serum albumin (BSA) at room temperature for 1h. Cells were washed again and treated with the following specific antibodies: FITC-conjugated rabbit anti-human C3c-complement (Dako, that recognizes C3c, part of C3 and C3b, 1:300 final dilution in Dapi 1 µg/mL); or rabbit anti-human complement C5b-9 complex (Calbiochem, 1:200 final dilution in PBS1X) followed by FITC-conjugated secondary antibody (Jackson ImmunoResearch Laboratories, 1:50 final dilution in 1 µg/mL Dapi); or goat anti-human C4 (Abcam, 1:100 final dilution in PBS1X) followed by Cy3-conjugated secondary antibody (Jackson ImmunoResearch Laboratories, 1:200 final dilution in Dapi 1 µg/mL); or FITC-conjugated anti-human IgG (Sigma Aldrich, 1:32 final dilution in 1 µg/mL Dapi); or mouse anti-human P-selectin (R&D System, 20 µg/mL final concentration in PBS1X), followed by Cy3-conjugated secondary antibody (Jackson ImmunoResearch Laboratories, 1:60 final dilution in 1 µg/mL Dapi); or rabbit anti-human vWF (Dako, 10 µg/mL final concentration in PBS1X), followed by Cy3-conjugated secondary antibody (Jackson ImmunoResearch Laboratories, 1:50 final dilution in 1 µg/mL Dapi).

The immunofluorescent staining on endothelial cell surface was evaluated with a fluorescence microscope (Axio Imager Z2, Zeiss). Fifteen non-overlapping fields per slide were acquired randomly using a computer-based image analysis system. The area occupied by the fluorescent staining was evaluated by automatic edge detection using built-in specific functions of the software Image J and expressed as pixel^2^ per field analyzed. The fields showing the highest and the lowest values were discarded and the mean was calculated on the remaining 13 fields. Results were expressed as pixel^2^ or as percentage of the staining relative to that obtained with NHS set at 100%, as specified.

For C4 and IgG deposits, sera from additional 15 healthy single subjects have been used to calculate the upper and the lower limit of a normal range, given as the mean ± 2SD of the percentages of the deposits obtained with sera from single subjects in respect to NHS run in parallel (**Supplementary Figures 2**, **4**).

For C3 and C5b-9 deposits, the upper and lower limit of a normal range, given as the mean ± 2SD of the percentage of stained area obtained with sera from single healthy subjects in respect to NHS, have been previously calculated both on unstimulated and on activated HMEC-1 and published (37, 38).

### Evaluation of thrombus formation under flow condition

The assay for the analysis of thrombus formation under flow condition was performed as previously described (37, 39).

At the end of the serum incubation step, HMEC-1 were washed and then perfused with heparinized (10 UI/mL heparin) whole blood (pre-labeled with the fluorescent dye mepacrine, quinacrine dihydrochloride 10 µM, Sigma Aldrich) obtained from healthy subjects. The perfusion with blood was performed in a thermostatic flow chamber (37°C) in which one surface of the perfusion channel was a glass slide seeded with a monolayer of HMEC-1 perfused at a constant flow rate of 1500 sec^-1^ (60 dynes/cm^2^) to mimic the shear stress condition in the microcirculation (40). To evaluate the role of Weibel-Palade bodies (WPB) exocytosis on thrombus formation on HMEC-1 pre-exposed with STEC-HUS serum, in selected experiments RalA – a GTPase central to the molecular machinery guiding WPB exocytosis – was inhibited by pre-incubating the cells for 16 hours with the specific RalA inhibitor BQU57 (10 µM, Merck). In each experiment, all slides were perfused with blood derived from the same sampling. After 3 minutes, the perfusion was stopped and the endothelial cell monolayer on each glass slide was dehydrated and fixed in acetone for 20 minutes.

Slides were examined under an inverted confocal laser-scanning microscope (either Leica CS SP8, Leica microsystems or LSM 510 Meta, Zeiss) at X200 magnification. Fifteen non-overlapping representative images per sample were acquired. The area occupied by thrombi was evaluated using Image J software as performed for immunofluorescence analysis, and expressed as pixel^2^ per field analyzed. The highest and lowest values were discarded and the mean was calculated on the remaining 13 fields. Results were expressed as percentage of stained area in respect to that obtained with NHS set at 100%.

The upper and lower limit of a normal range, given as the mean ± 2SD of the percentage of stained area obtained with single sera from 10 healthy subjects in respect to NHS, have been previously calculated and published (37).

### Scanning electron microscopy

For scanning electron microscopy (SEM) analysis, HMEC-1 were cultured on 60x20 mm plastic slides (Thermanox; Nunc) and used when confluent. Thrombus formation experiments were performed as described above except that the blood was un-labeled. At the end of the 3 minutes of blood perfusion, slides were pre-fixed for 1 hour in 2.5% glutaraldehyde (buffered with 0.1 M sodium cacodylate buffer, pH 7.4). The slides were then repeatedly washed in cacodylate buffer and post-fixed in 1% osmium tetroxide for 1 hour. Fixed specimens were dehydrated with increasing concentrations of alcohol and then were dehydrated in pure HMDS (Polyscience) (twice for 0.5 hours). Samples were then mounted on stubs and coated with gold in a sputter coater (Agar Scientific Ltd, Stansted, England). Coated specimens were observed through SEM using secondary electron detection (Supra 55, Zeiss, Oberkochen, Germany). Acceleration voltage was set to 1.5-2.0 kV and enlargement up to 350 kx.

### Circulating complement component assays

The determination of circulating complement C3 and C4 levels in serum was determined by nephelometry (normal range, defined as mean ± 2SD, for C3: 83–180 mg/dL, n=50; for C4: 10–40 mg/dL).

The measurement of sC5b-9 levels in EDTA plasma was performed using MicroVue sC5b-9 Plus EIA (SC5b-9 Plus; Quidel). The normal range of plasma sC5b-9 in our laboratory is 110–335 ng/mL (n=40).

### Statistical analyses

The normality of value distribution was assessed using the D’Agostino-Pearson’s test or Shapiro-Wilk test, as appropriate. For comparisons between two groups, significant differences were determined using Student’s t test (either for unpaired or for paired samples) or using Mann-Whitney test, as appropriate. Comparisons of three or more groups were performed with one-way analysis of variance followed by *post hoc* Tukey’s multiple comparison test or Holm-Šídák’s multiple comparisons test, as appropriate. Pearson or Spearman correlation coefficient, as appropriate, were used to correlate continuous variables. The specific statistical test used for each analysis is reported in the corresponding figure legend.

P values of less than 0.05 were considered statistically significant. All data were analyzed using GraphPad Prism 9.5.0 (730) software.

## Results

### Serum from STEC-HUS patients in acute phase induces C3 deposition on microvascular endothelial cells

Thirty-seven STEC-HUS patients, of whom 33 were children, were recruited during the acute phase of the disease. The clinical characteristics of individual patients are summarized in **Table 1**. Analysis of circulating C3 and sC5b-9 levels (measured in 24 and 27 patients, respectively) showed evidence of C3 consumption in 10 patients, while sC5b-9 levels were above the normal range in 18 of 27 patients.

**Table 1:** Characteristics, clinical and complement parameters of STEC-HUS patients recruited during the acute phase of disease.

Patient no.	Age of onset (years)	Sex	*Ex vivo* C5b-9 formation (% of NHS)		Clinical parameters^a^						Circulating complement profile^d^		
					Platelets, x10^3^/µL	Hemoglobin, g/dL	LDH, IU/L	Hp, mg/dL	sCr, mg/dL	*E. coli* serotype	C3, mg/dL	C4, mg/dL	sC5b-9, ng/mL
	Mean = 10 (0-78)	22♀, 15♂	Unstimulated	ADP-activated	Ref range 150-400	Ref range^b^	Ref range 266-500	Ref range 49-246	Ref range^c^		Ref range 83-180	Ref range 10-40	Ref range 110-335
**Patient 1**	1	F	209	204	46	11.8	6762	7	3.43	O26	61	14	499
**Patient 2**	0	F	334	321	46	7	3112	<1	2.70	nd	83	8	214
**Patient 3**	2	F	425	174	nd	nd	nd	nd	nd	O26	nd	nd	nd
**Patient 4**	65	F	258	276	44	9.8	719	<7.75	6.70	nd	90	16	nd
**Patient 5**	64	F	200	210	73	8	308	<1	1.71	nd	71	9	186
**Patient 6**	1	F	231	242	55	13	1044	<1	0.29	O126	61	6	349
**Patient 7**	12	M	182	229	16	9.4	294	0.15	0.61	O63:H6	118	27	179
**Patient 8**	1	F	166	178	389	8.2	763	<1	0.31	O26	78	9	351
Patient 9	3	M	247	232	76	8.8	1376	nd	0.83	O26	75	17	235
**Patient 10**	3	F	358	238	58	9.8	2131	<1	1.89	nd	57	4	nd
**Patient 11**	1	M	386	232	49	7.4	1787	<1	1.57	O157:H7	117	24	nd
**Patient 12**	2	M	299	281	80	6	2640	<1	1.22	O26:H11	137	16	nd
Patient 13	1	M	271	233	136	8.9	1160	<1	0.51	O26:H11	98	20	366
Patient 14	4	F	nd	nd	35	9.3	545	<1	1.44	O157:H7	96	17	179
Patient 15	5	M	199	278	92	7.6	1259	<1	1.53	O26	94	28	415
Patient 16	1	F	218	286	nd	nd	nd	nd	nd	nd	nd	nd	480
Patient 17	78	M	193	194	37	8	4425	11.4	5.45	nd	nd	nd	512
Patient 18	8	M	nd	469	10	10	nd	nd	5.00	nd	nd	nd	347
Patient 19	3	M	nd	475	41	6.3	7019	1	8.57	O26	88	19	436
Patient 20	2	F	256	277	160	8	4620	1.5	3.70	O111	nd	nd	269
Patient 21	5	M	162	169	145	7.7	890	<0.08	0.67	nd	50	17	1039
Patient 22	45	F	226	231	28	8.9	1870	6	1.43	nd	108	26	nd
Patient 23	9	F	203	216	nd	nd	nd	nd	nd	O145	nd	nd	nd
Patient 24	8	F	153	188	70	8.9	5325	nd	3.99	O157	nd	nd	839
Patient 25	17	F	209	227	138	12.8	nd	nd	4.30	nd	76	7	486
Patient 26	3	F	163	216	77	9.5	3907	nd	1.64	nd	74	11	884
Patient 27	2	F	291	301	66	8.5	2989	nd	3.12	nd	nd	nd	637
Patient 28	6	F	538	348	42	7.5	3981	nd	10.24	nd	nd	nd	951
Patient 29	5	M	251	287	68	6.8	3915	<1	2.18	O183:H9	141	24	637
Patient 30	3	F	141	178	165	12.3	1919	<1	0.54	O26	148	20	181
Patient 31	3	F	186	193	nd	nd	4000	nd	nd	nd	52	7	252
Patient 32	3	M	198	217	51	7.7	3368	<0.08	3.00	nd	83	18	726
Patient 33	4	M	195	205	nd	nd	nd	nd	nd	O26	nd	nd	nd
Patient 34	2	F	199	209	36	10.2	5014	nd	2.68	nd	nd	nd	575
Patient 35	10	M	190	183	nd	nd	nd	nd	nd	nd	nd	nd	nd
Patient 36	1	F	225	351	nd	nd	nd	nd	nd	O111	nd	nd	225
Patient 37	8	M	179	296	nd	nd	nd	nd	nd	nd	89	19	nd

Hp, haptoglobin; LDH, lactate dehydrogenase; sCr, serum creatinine; nd, not determined.

a Clinical data in the table are those recorded the same days that complement parameters were evaluated.

b Normal range: 10.8-12.5 g/dL for children aged 5 months to 1 year; 11.5-13.5 g/dL for children aged 1 to 5 years; 12-14.5 g/dL for children aged 5 to 10 years; 12–15 g/dL for adult female; 13–16 g/dL for adult male.

c Normal range: 0.3-0.5 mg/dL for children <1–5 years; 0.5-0.8 mg/dL for children aged 5 to 10 years; 0.5-1.2 mg/dL for children >10 years and adults.

d C3 and C4 measured in serum; sC5b-9 measured in plasma.

Bold: patients studied both during the acute phase and at follow-up.

**Figure 1:**
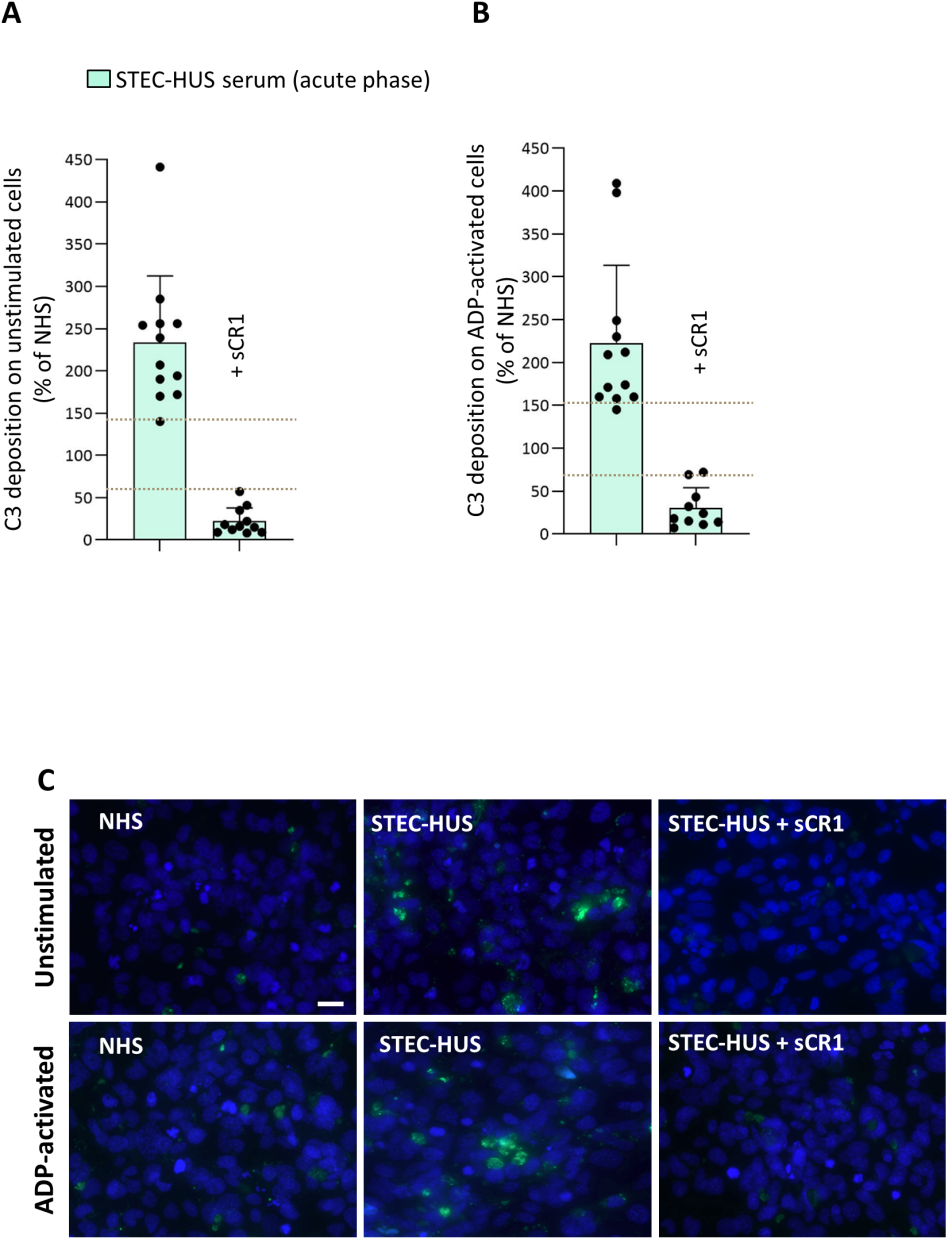
*Ex-vivo* serum-induced C3 deposits on unstimulated and ADP-activated microvascular endothelial cells (HMEC-1) in patients with acute STEC-HUS. C3 deposition after incubation of unstimulated **(A)** and ADP-activated **(B)** HMEC-1 with serum from patients with acute STEC-HUS (n = 12). The addition of the pan complement inhibitor sCR1 (150 µg/mL) to the patients’ serum (n = 11 on unstimulated and n = 10 on ADP-activated cells, depending on serum availability) completely inhibited abnormal C3 deposition. Data are expressed as mean ± SD of percentages of serum-induced C3 deposition in respect to a pool of control sera (normal human serum, NHS), run in parallel in each experiment and set as 100%. Circles indicate single patients’ data. Horizontal dashed lines indicate upper and lower limits of the normal range (37). **(C)** Representative fluorescent microscopy images of C3 staining (in green) on unstimulated and ADP-activated HMEC-1 exposed to NHS, or to serum from a STEC-HUS patient collected during the acute phase in the presence and in the absence of sCR1 (original magnification X400). Scale bar: 20 µm. The blue color indicates the DAPI staining of cell nuclei.

**Figure 2:**
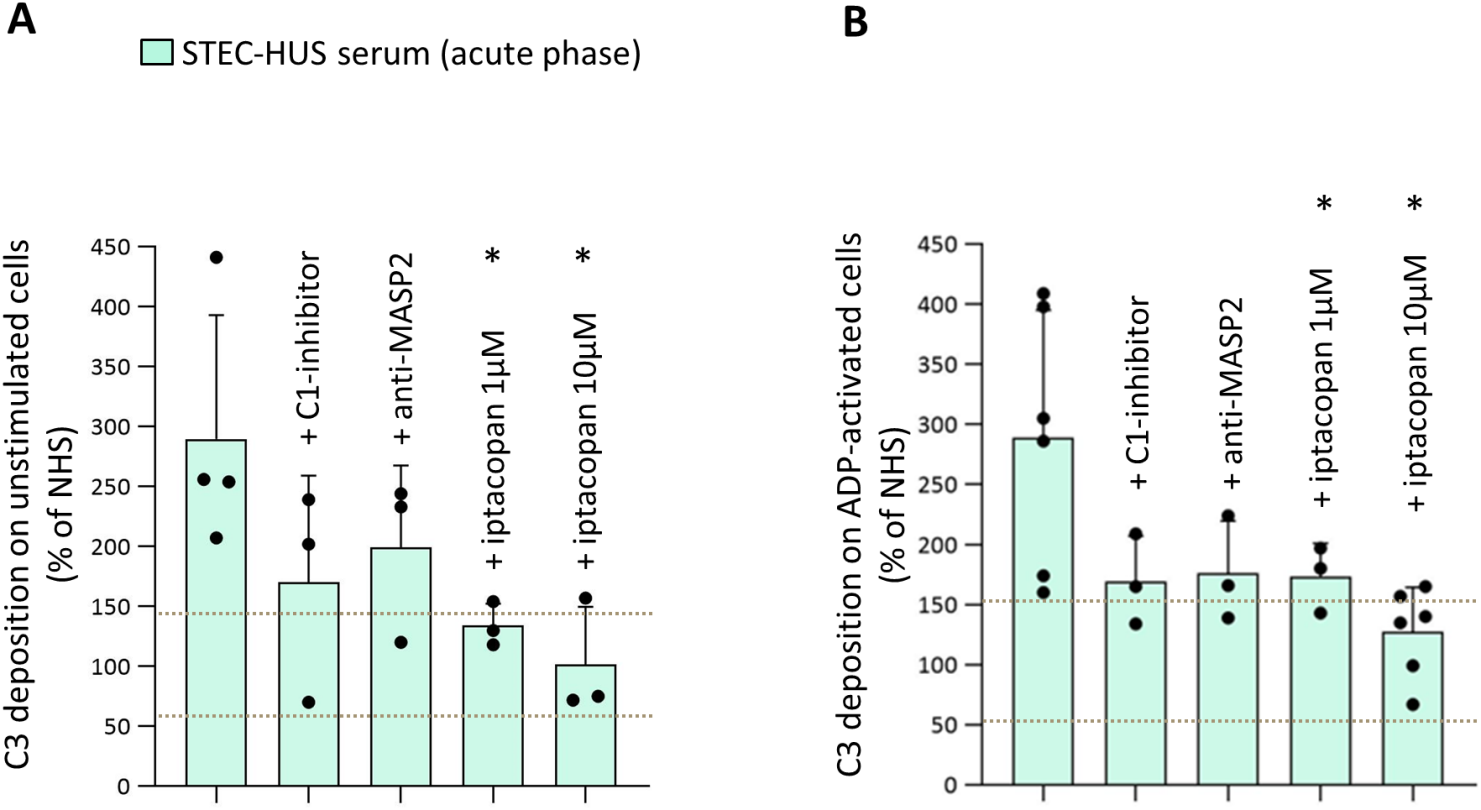
Effect of complement inhibitors on STEC-HUS serum-induced C3 deposition on unstimulated and ADP-activated microvascular endothelial cells (HMEC-1). C3 deposition after incubation of unstimulated **(A)** and ADP-activated **(B)** HMEC-1 with serum from patients with acute STEC-HUS, in the presence or in the absence of different complement inhibitors (C1-inhibitor, 36 µg/mL; anti-MASP2, 2 µg/mL; factor B inhibitor, iptacopan, 1 µM or 10 µM). Data are expressed as mean ± SD of percentages of serum-induced C3 deposition in respect to a pool of control sera (normal human serum, NHS), run in parallel in each experiment and set as 100%. Circles indicate single patients’ data. Horizontal dashed lines indicate upper and lower limits of the normal range (37). The addition of iptacopan at both the concentrations tested in the patients’ serum significantly prevented the C3 deposition, both on unstimulated and on ADP-activated cells. *P < 0.05, vs STEC-HUS alone (paired Student’s t test between data of C3 deposits induced by patients’ sera and data obtained by the same patients’ sera added with a specific inhibitor).

The analysis of serum-induced C3 deposition was performed in 12 patients on both unstimulated and ADP-activated HMEC-1, the latter mimicking a condition of endothelial perturbation (**Figure 1**). On both unstimulated and activated HMEC-1, incubation with serum from acute STEC-HUS induced a 2-3-fold elevated C3 deposition, compared to that observed on cells exposed to a pool of control sera from healthy subjects (NHS), run in parallel in each experiment and set as 100% (**Figures 1A-C**). C3 deposition exceeded the upper limit of the control range with sera from 11 out of 12 patients (**Figure 1**) and was completely prevented by sCR1 added to patients’ sera, indicating that the staining was specifically related to complement activation (**Figures 1A-C**). In selected experiments, serum-induced C3 deposition on HMEC-1 was analyzed in the presence of a C1-inhibitor, an anti-MASP2 blocking antibody, or a FB inhibitor (LNP023, iptacopan), to block the classical, the lectin or the alternative pathway, respectively (**Figures 2A, B**). Results showed that both the C1-inhibitor and the anti-MASP2 antibody only partially reduced STEC-HUS serum-induced C3 deposits, both on unstimulated and activated HMEC-1. At variance, iptacopan significantly reduced *ex-vivo* STEC-HUS serum-induced C3 deposits, which fell into the normal range with the 10 µM dose (**Figures 2A, B**).

To verify whether the classical pathway (CP) and/or the lectin pathway (LP) were indeed activated on HMEC-1 by STEC-HUS serum, the experiments were repeated with C4 staining as reading. Only 3 out of 8 of the analyzed STEC-HUS sera induced C4 deposition over normal range on both unstimulated and activated HMEC-1 (**Supplementary Figures 2**, **3**), so that mean values (pixel^2^/field) did not significantly differ from C4 staining on cells incubated with NHS. At variance, significantly elevated C4 staining was observed on cells exposed to sera from four SLE patients taken as positive controls of classical pathway activation (**Supplementary Figure 3**).

Consistent with C4 staining results, serum from only 1 out of 6 STEC-HUS patients induced abnormal IgG deposition on both unstimulated and activated HMEC-1, at variance with intense IgG staining observed on HMEC-1 exposed to serum from patients with SLE (n = 3) taken as a positive control (**Supplementary Figures 4**, **5**).

### Serum from STEC-HUS patients in acute phase induces C5b-9 formation on microvascular endothelial cells

**Figure 3:**
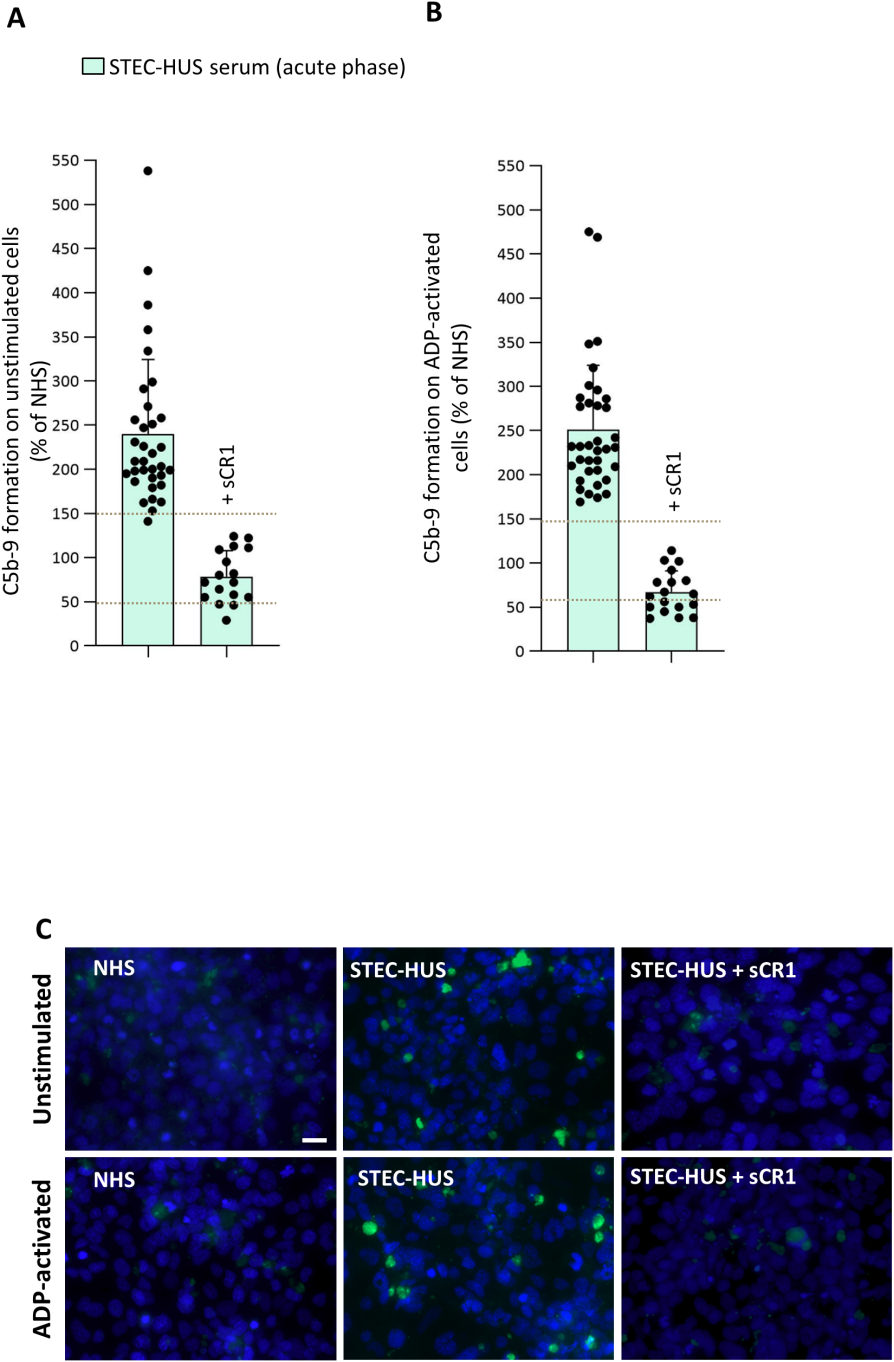
*Ex-vivo* serum-induced C5b-9 formation on unstimulated and ADP-activated microvascular endothelial cells (HMEC-1) in patients with acute STEC-HUS. C5b-9 formation after incubation of unstimulated **(A)** and ADP-activated **(B)** HMEC-1 with serum from patients with acute STEC-HUS (n = 34 on unstimulated and n = 36 on ADP-activated HMEC-1). The addition of sCR1 to the patients’ serum (150 µg/mL, n = 17 on unstimulated and n = 18 on ADP-activated cells, depending on serum availability) completely inhibited abnormal C5b-9 formation. Data are expressed as mean ± SD of percentages of serum-induced C5b-9 formation in respect to a pool of control sera (normal human serum, NHS), run in parallel in each experiment and set as 100%. Circles indicate single patients’ data. Horizontal dashed lines indicate upper and lower limits of the normal range (38). **(C)** Representative fluorescent microscopy images of C5b-9 staining (in green) on resting and ADP-activated HMEC-1 exposed to NHS, or to serum from a STEC-HUS patient collected during the acute phase, in the presence and in the absence of sCR1 (original magnification X400). Scale bar: 20 µm. The blue color indicates the DAPI staining of cell nuclei.

**Figure 4:**
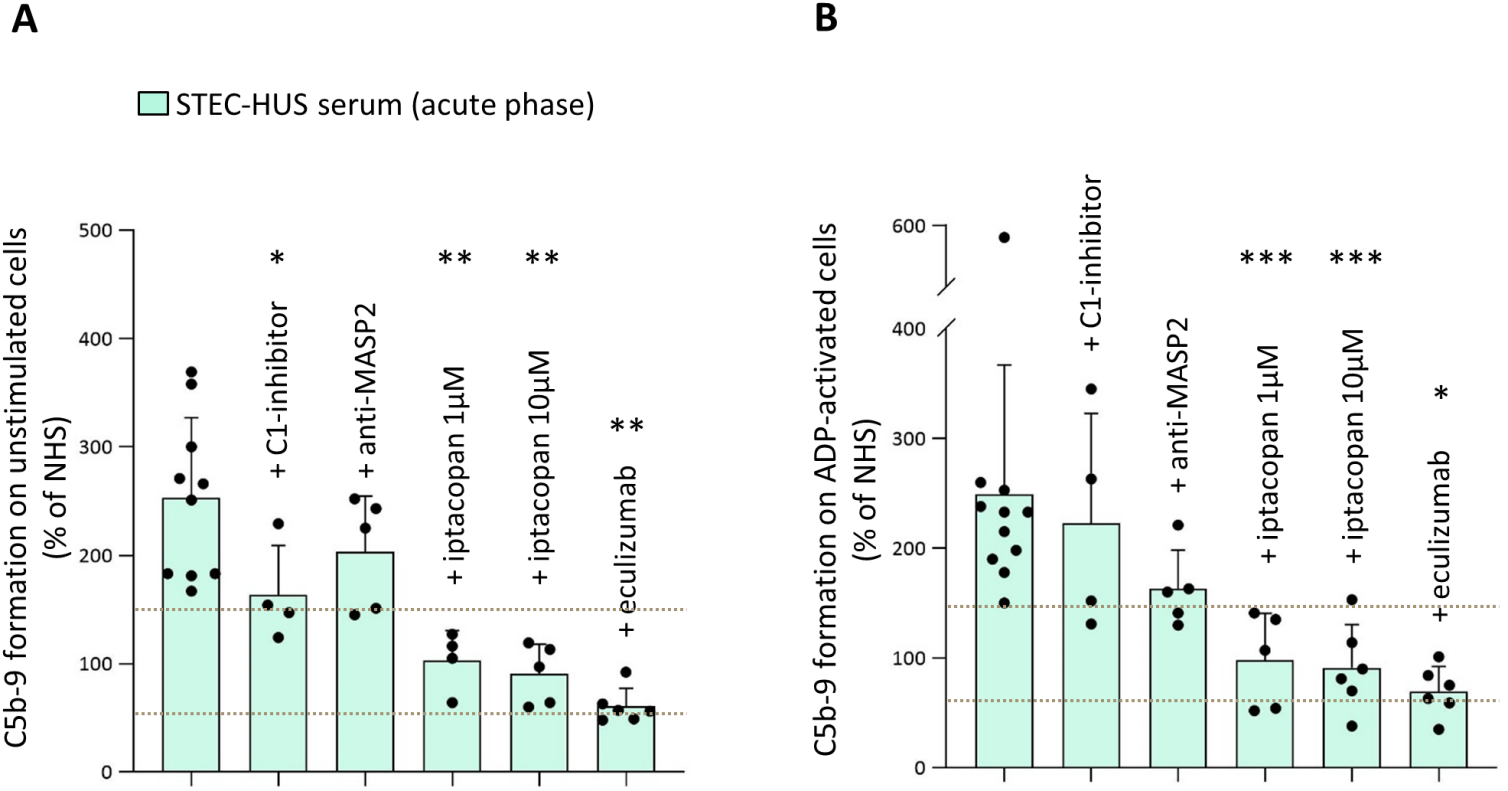
Effect of complement inhibitors on STEC-HUS serum-induced C5b-9 formation on unstimulated and ADP-activated microvascular endothelial cells (HMEC-1). C5b-9 formation after incubation of unstimulated **(A)** and ADP-activated **(B)** HMEC-1 with serum from patients with acute STEC-HUS, in the presence or in the absence of different complement inhibitors (C1-inhibitor, 36 µg/mL; anti-MASP2, 2 µg/mL; factor B inhibitor, iptacopan, 1 µM or 10 µM; eculizumab, 100 µg/mL). Data are expressed as mean ± SD of percentages of serum-induced C5b-9 formation in respect to a pool of control sera (normal human serum, NHS), run in parallel in each experiment and set as 100%. Circles indicate single patients’ data. Horizontal dashed lines indicate upper and lower limits of the normal range (38). The addition of iptacopan at both concentrations or of eculizumab to the patients’ serum significantly prevented the C5b-9 formation, both on unstimulated and on ADP-activated cells. *P < 0.05, **P < 0.01, ***P < 0.001 vs STEC-HUS alone (paired Student’s t test between data of C5b-9 formation induced by patients’ sera and data obtained by the same patients’ sera added with a specific inhibitor).

Formation of C5b-9 was evaluated on both unstimulated (n = 34) and ADP-activated (n = 36) HMEC-1 exposed to serum from STEC-HUS patients collected during the acute phase. Excessive C5b-9 formation on unstimulated HMEC-1 and ADP-activated HMEC-1 was observed with sera from 33 of 34 and 36 of 36 acute STEC-HUS patients, respectively (**Figures 3A-C**). In line with results of C3 deposition, addition of sCR1 to patients’ sera completely normalized C5b-9 formation on HMEC-1 (**Figures 3A-C**). Serum-induced C5b-9 formation on activated HMEC-1 did not correlate with either circulating levels of C3 (n = 24, P = 0.65) or sC5b-9 (n = 25, P = 0.64) (**Supplementary Figure 6**). Selected experiments were repeated in the presence of a C1-inhibitor, an anti-MASP2 blocking antibody, iptacopan, or eculizumab (**Figures 4A, B**). The C1-inhibitor significantly decreased C5b-9 formation induced by STEC-HUS serum on unstimulated HMEC-1, but had no significant effect on ADP-activated cells. The anti-MASP2 antibody had no significant effect on both unstimulated and activated cells (**Figures 4A, B**). At variance, the addition of iptacopan at both concentrations to patients’ sera fully normalized serum-induced *ex-vivo* C5b-9 formation on both unstimulated and ADP-activated cells (**Figures 4A, B**). Of relevance, the inhibitory effect of iptacopan on C5b-9 formation was comparable to that observed with eculizumab (**Figures 4A, B**).

### *Ex-vivo* serum-induced C5b-9 formation on microvascular endothelial cells correlates with serum creatinine and LDH levels in acute STEC-HUS

It was then investigated whether serum-induced *ex-vivo* C5b-9 formation on endothelial cells was associated with indices of disease severity, specifically serum creatinine values, which reflect renal function impairment, and plasma levels of LDH, marker of tissue/organ injury and hemolysis. A positive correlation was found between values of serum-induced C5b-9 formation on activated cells and levels of both serum creatinine (**Supplementary Figure 7A**), and plasmatic LDH (**Supplementary Figure 7B**). At variance, circulating levels of C3 and sC5b-9 did not correlate with either clinical parameter (**Supplementary Figure 8**).

### Serum from patients with STEC-HUS in acute phase induces thrombus formation on microvascular endothelial cells

**Figure 5:**
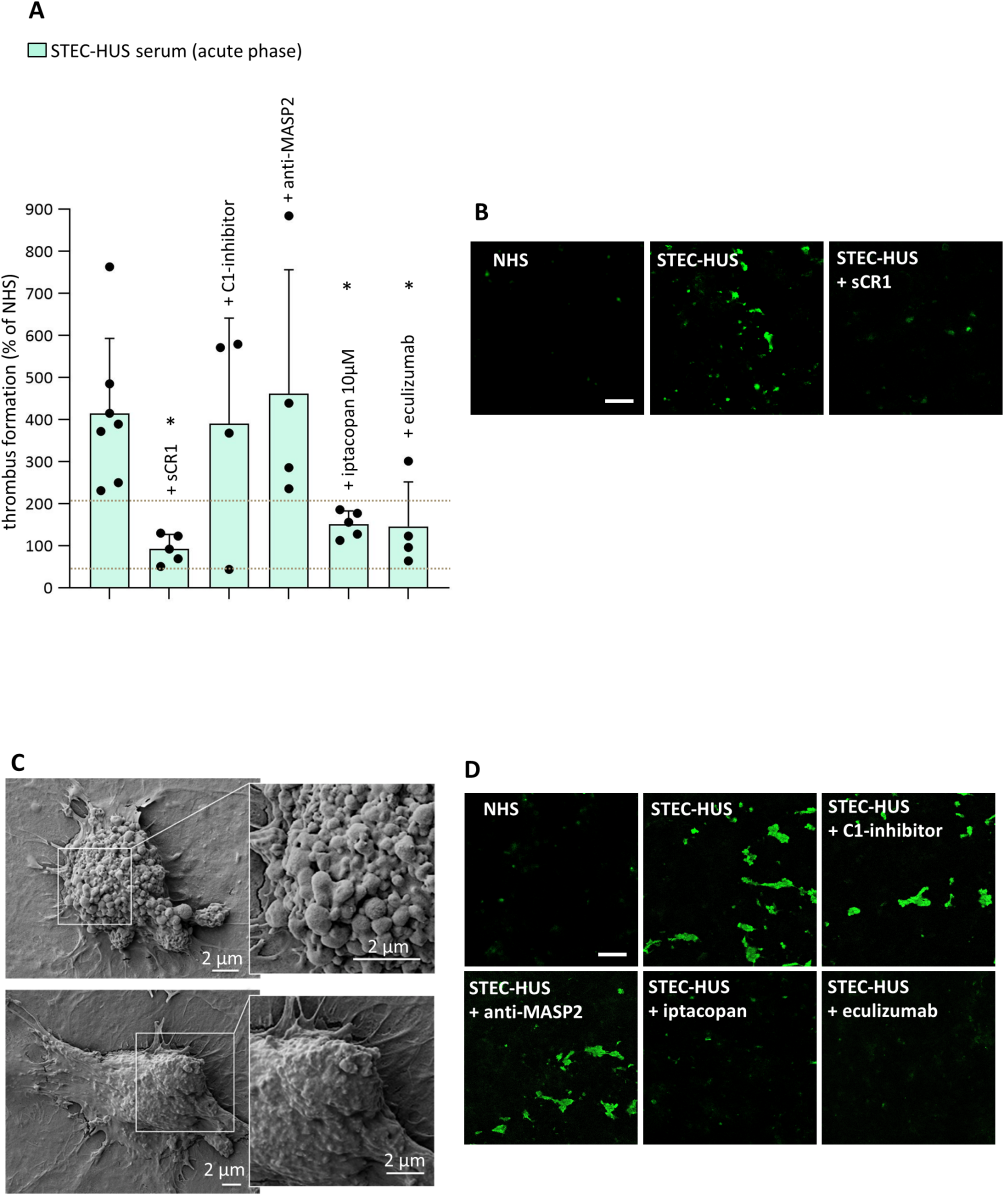
*Ex-vivo* serum-induced thrombus formation on ADP-activated microvascular endothelial cells (HMEC-1) in acute STEC-HUS. **(A)** Endothelial surface area covered by thrombi on ADP-activated HMEC-1 exposed to serum from STEC-HUS patients collected during the acute phase of the disease, in the presence or in the absence of different complement inhibitors (C1-inhibitor, 36 µg/mL; anti-MASP2, 2 µg/mL; factor B inhibitor, iptacopan, 10 µM; eculizumab, 100 µg/mL). Data are expressed as mean ± SD of percentages of serum-induced thrombus formation in respect to a pool of control sera (normal human serum, NHS), run in parallel in each experiment and set as 100%. Circles indicate single patients’ data. Horizontal dashed lines indicate upper and lower limits of the normal range (37). The addition of sCR1 or iptacopan or eculizumab in the patients’ serum significantly reduced thrombus formation. *P < 0.05 vs STEC-HUS alone (paired Student’s t test between data of thrombus formation induced by patients’ sera and data obtained by the same patients’ sera added with a specific inhibitor). **(B)** Representative confocal microscopy images (original magnification X200) of thrombus formation (thrombus staining, in green) on ADP-activated HMEC-1 exposed to NHS or to serum from a STEC-HUS patient collected during the acute phase, in the presence or in the absence of sCR1. Scale bar: 100 µm. **(C)** Representative images of the ultrastructure of aggregates of platelets adhered on HMEC-1 pre-exposed to serum from a STEC-HUS patient in the acute phase of disease and then perfused with whole blood, evaluated with scanning electron microscopy. Insets show high-power view of the same platelet aggregate. Scale bar: 2 µm. **(D)** Representative confocal microscopy images (original magnification X200) of thrombus formation (thrombus staining, in green) on ADP-activated HMEC-1 exposed to NHS or to serum from a STEC-HUS patient collected during the acute phase, in the presence or in the absence of different complement inhibitors. Scale bar: 100 µm.

It was then evaluated whether acute STEC-HUS serum caused loss of endothelial thromboresistance in HMEC-1 and the role of complement. As shown in **Figure 5**, the area covered by thrombi was 2-7-fold larger on HMEC-1 pre-incubated with patients’ sera and then perfused with control whole blood, as compared with HMEC-1 pre-incubated with NHS, which was set as 100% (**Figures 5A, B**). Notably, all analyzed STEC-HUS sera induced a thrombus area above the upper limit of the normal range. Scanning electron microscopy evaluation of HMEC-1 pre-exposed to STEC-HUS serum and then perfused with blood, documented the attachment of platelets to the endothelial cell monolayer to form well organized aggregates (**Figure 5C**). The formation of thrombi was completely inhibited by adding sCR1 to patients’ sera, indicating that it was dependent on complement activation (**Figures 5A, B**).

Iptacopan and eculizumab fully prevented the abnormal formation of thrombi induced *ex-vivo* by STEC-HUS serum on endothelial cells, whereas the C1-inhibitor and the anti-MASP2 antibody had no effect (**Figures 5A, D**). These data indicate that the pro-thrombotic effect of STEC-HUS serum mainly relies on complement activation selectively via the alternative pathway.

### The prothrombotic effects of STEC-HUS serum are associated with exocytosis of Weibel-Palade bodies from endothelial cells

**Figure 6:**
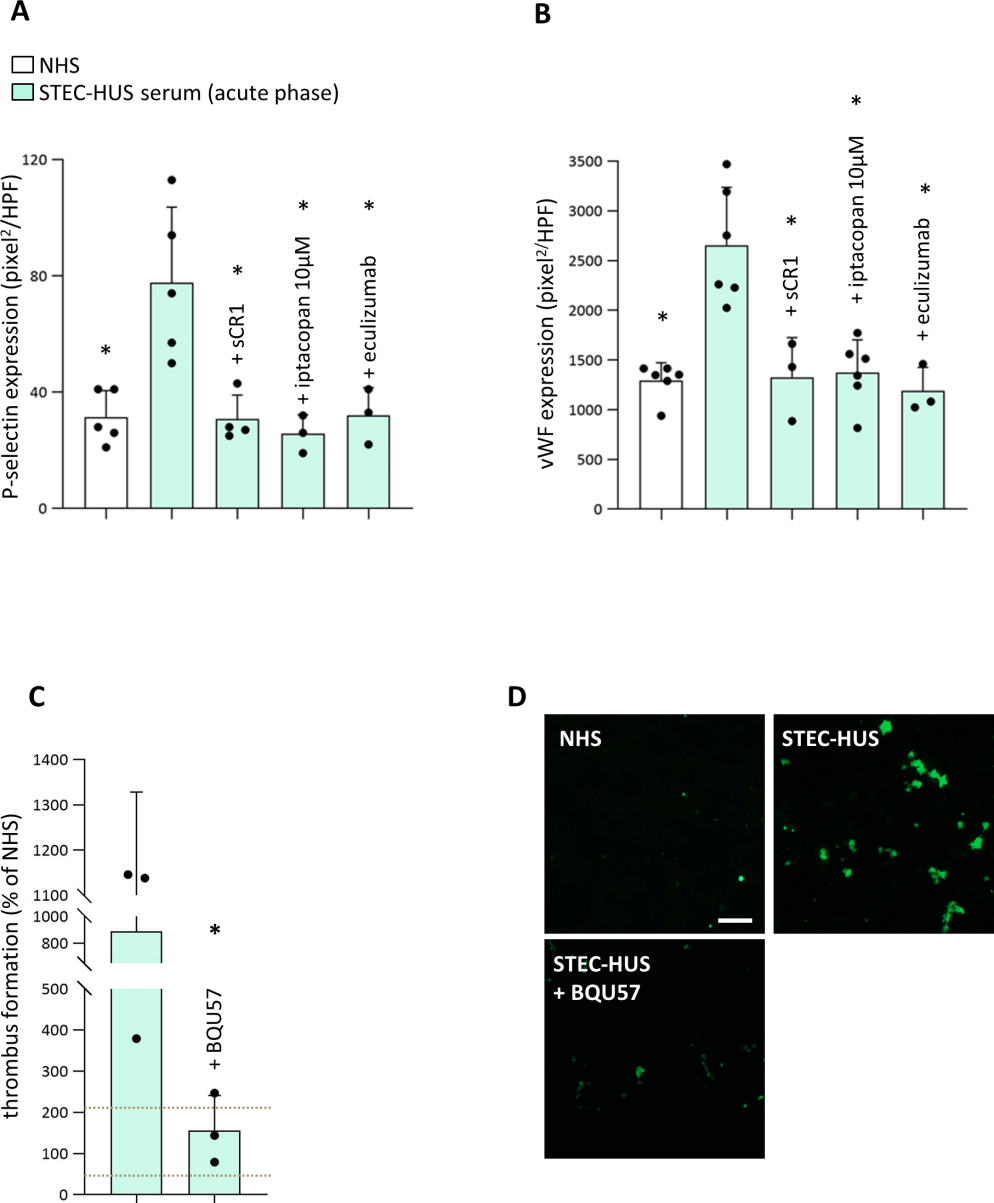
Prothrombotic effects of STEC-HUS serum are dependent on WPB exocytosis from endothelial cells. P-selectin **(A)** and vWF **(B)** expression on unstimulated HMEC-1 exposed to serum from patients with acute STEC-HUS (n = 5 for P-selectin experiments; n = 6 for vWF experiments), in the presence or in the absence of different complement inhibitors (sCR1, 150 µg/mL; factor B inhibitor, iptacopan,10 µM; eculizumab 100 µg/mL) or to a pool of control sera (normal human serum, NHS), run in parallel. Results are shown as pixel^2^/high-power field (HPF) of stained surface area. Data are mean ± SD. Circles represent single patients’ data. The addition of either sCR1, or iptacopan or eculizumab to patient’s serum significantly decrease both P-selectin and vWF expression induced by patient’s serum alone. *P < 0.05 vs STEC-HUS serum alone (ANOVA, followed by Tukey’s multiple comparisons test for data of P-selectin expression and by Holm-Šídák’s multiple comparisons test for data of vWF expression). **(C)** Endothelial surface area covered by thrombi on ADP-activated HMEC-1 exposed to serum from STEC-HUS patients collected during the acute phase of the disease and then perfused with whole blood. Before the experiments, HMEC-1 were left for 16 hours with medium added or not with the RalA inhibitor BQU57 (10 µM). Data are expressed as mean ± SD of percentages of serum-induced thrombus formation in respect to a pool of control sera (normal human serum, NHS), run in parallel in each experiment and set as 100% (n = 3 independent experiments). Circles indicate single patients’ data. Horizontal dashed lines indicate upper and lower limits of the normal range (37). *P < 0.05 (paired Student’s t test). **(D)** Representative confocal microscopy images (original magnification X200) of experiments of thrombus formation (green staining) relative to **Figure 6C**. Scale bar: 100 µm.

To study the mechanism(s) underlying the prothrombogenic action of STEC-HUS serum, P-selectin and vWF expression was evaluated on cell surface of HMEC-1 exposed to serum from STEC-HUS patients with active disease. As compared with HMEC-1 exposed to NHS, cells incubated with STEC-HUS serum showed a significant increase in cell surface expression of P-selectin that was prevented in the presence of either sCR1, or iptacopan or eculizumab (**Figure 6A**). As shown in **Figure 6B**, incubation with STEC-HUS serum induced also significantly higher expression of vWF vs. NHS, which suggests that STEC-HUS serum stimulated the release of Weibel-Palade bodies (WPB) content from HMEC-1. Addition of sCR1, iptacopan or eculizumab to STEC-HUS serum significantly reduced the vWF-stained area on HMEC-1 (**Figure 6B**).

To assess the role of the WPB exocytosis in the pro-thrombotic effect of STEC-HUS serum, experiments of thrombus formation under flow conditions were repeated with HMEC-1 preincubated with BQU57, a specific inhibitor of RalA, which is a small GTPase central to the molecular machinery guiding WPB exocytosis (41–43). Pre-incubation of HMEC-1 with BQU57 fully prevented thrombus formation on the surface of HMEC-1 exposed to sera from STEC-HUS patients with active disease (**Figures 6C, D**).

### C5b-9 formation induced on HMEC-1 by serum from STEC-HUS patients at follow-up

A total of 24 STEC-HUS patients were studied after hospital discharge (≥ 16 days from the acute episode, range 16–756 days). On the basis of clinical parameters (summarized in **Table 2**), 9 patients were classified as being in full remission, defined as normalization of both hematological parameters and renal function. Fifteen patients were classified as being in partial remission, defined by either normal hematological parameters with residual renal dysfunction or no normalization of at least one hematological parameter. Analysis of circulating C3 and sC5b-9 levels (measured in 10 and 14 patients, respectively) showed no evidence of C3 consumption in any patient, while sC5b-9 levels were above the normal range in 3 of 14 patients.

**Table 2:** Characteristics, clinical and complement parameters of STEC-HUS patients analyzed at follow-up.

Patient no.	Age at follow-up (years)	Sex	*Ex vivo* C5b-9 formation (% of NHS)	Clinical parameters^a^						Circulating complement profile^d^			Notes
				Platelets, x10^3^/µL	Hemoglobin, g/dL	LDH, IU/L	Hp, mg/dL	sCr, mg/dL	*E. coli* serotype	C3, mg/dL	C4, mg/dL	sC5b-9, ng/mL	
	Mean = 12 (1-65)	13♀, 11♂	ADP-activated	Ref range 150-400	Ref range^b^	Ref range 266-500	Ref range 49-246	Ref range^c^		Ref range 83-180	Ref range 10-40	Ref range 110-335	
Patient 45	21	F	117	209	13.2	422	162	0.86	nd	118	17	205	
**Patient 11**	1	M	57	507	12.3	330	112	0.24	O157	nd	nd	nd	
**Patient 12**	1.5	M	82	390	12.5	344	nd	0.38	O26	nd	nd	212	
Patient 46	4	M	140	297	13.0	394	nd	0.33	O26/O111	nd	nd	nd	
**Patient 10**	3	F	144	209	12.2	425	40	0.19	nd	144	18	283	
Patient 50	4	F	154	336	11.9	477	281	0.21	O26	135	24	410	
**Patient 7**	14	M	254	194	13.8	nd	62	0.50	O63	nd	nd	142	
**Patient 6**	3	F	278	294	12.9	471	62	0.35	O126	nd	nd	305	
Patient 44	2	M	180	268	13.0	nd	57	0.51	nd	nd	nd	nd	
Patient 42	5	M	175	224	12.0	212	92	9.00	O111	nd	nd	163	Dialysis
Patient 47	13	F	79	363	10.4	368	158	2.20	O157	nd	nd	nd	CKD
**Patient 8**	2	F	75	488	11.1	549	106	0.37	O26	95	8	nd	
Patient 48	7	F	239	397	10.0	249	14	0.49	O26	93	41	156	
Patient 49	13	F	163	323	10.6	455	100	5.34	O111	83	21	337	CKD
**Patient 3**	2	F	162	317	11.8	598	14	0.40	O26	104	23	nd	
Patient 39	2	M	168	331	13.0	296	26	0.36	O26	nd	nd	nd	Hypertension
**Patient 5**	65	F	162	190	13.2	535	106	1.53	nd	126	22	186	CKD
**Patient 1**	3	F	285	335	13.9	545	145	0.67	O26	nd	nd	344	
Patient 41	2	M	163	224	13.0	nd	8	0.34	O157	nd	nd	nd	
Patient 40	6	M	200	447	11.7	335	9	0.48	O26	nd	nd	nd	
Patient 43	1	M	359	320	12.6	760	56	0.23	nd	103	21	214	Hypertension
Patient 38	58	M	256	226	12.4	nd	93	0.77	O157	nd	nd	225	
**Patient 4**	65	F	318	210	14.0	601	5	1.21	nd	94	16	295	
**Patient 2**	1	F	203	530	12.0	657	90	0.35	nd	nd	nd	nd	

Hp, haptoglobin; LDH, lactate dehydrogenase; sCr, serum creatinine; CKD, chronic kidney disease; nd, not determined. In red: clinical parameters that are outside the age-specific normal range.

a Clinical data in the table are those recorded the same days that complement parameters were evaluated.

b Normal range: 10.8-12.5 g/dL for children aged 5 months to 1 year; 11.5-13.5 g/dL for children 1–5 years; 12-14.5 g/dL for children aged 5 to 10 years; 12–15 g/dL for adult female; 13–16 g/dL for adult male.

c Normal range: 0.3-0.5 mg/dL for children <1–5 years; 0.5-0.8 mg/dL for children aged 5 to 10 years; 0.5-1.2 mg/dL for children >10 years and adults.

d C3 and C4 measured in serum; sC5b-9 measured in plasma.

Bold: patients studied both during the acute phase and at follow-up.

**Figure 7:**
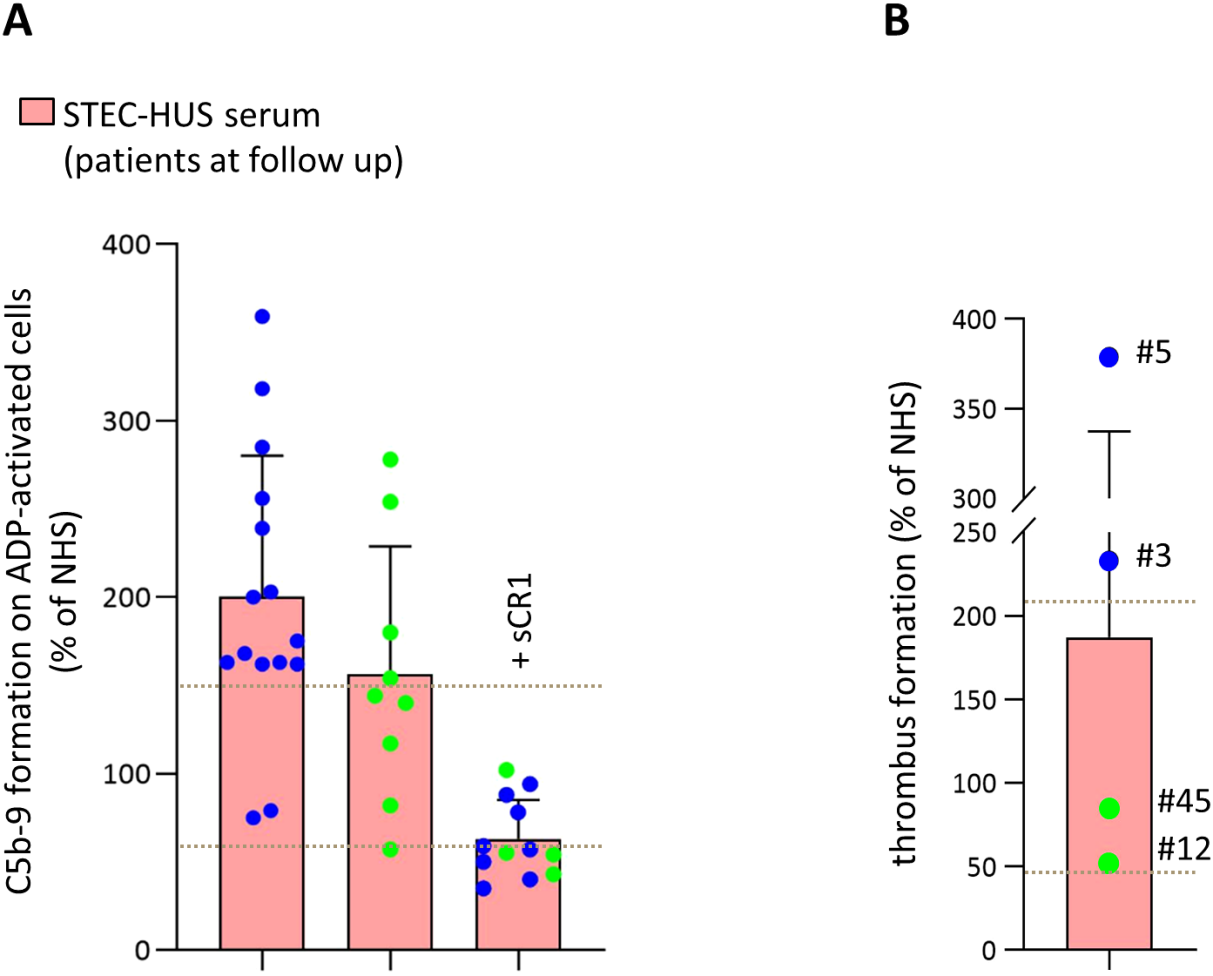
*Ex-vivo* serum-induced C5b-9 formation on ADP-activated microvascular endothelial cells (HMEC-1) in STEC-HUS patients analyzed at follow up after the resolution of STEC infection. **(A)** C5b-9 formation after incubation of ADP-activated HMEC-1 with serum from patients analyzed during follow-up, after resolution of STEC infection (n = 24). Depending on serum availability, the inhibitor sCR1 was used for n = 12. Data are expressed as mean ± SD of percentages of serum-induced C5b-9 formation in respect to a pool of control sera (normal human serum, NHS), run in parallel in each experiment and set as 100%. Circles indicate single patients’ data. Green and blue circles identify patients who were in full or in partial remission, respectively. Horizontal dashed lines indicate upper and lower limits of the normal range (38). **(B)** Endothelial surface area covered by thrombi on ADP-activated HMEC-1 exposed to serum from STEC-HUS patients #12, #45, #3, and #5. Data are expressed as mean ± SD of percentages of serum-induced thrombus formation in respect to a pool of control sera (normal human serum, NHS), run in parallel in each experiment and set as 100%. Circles indicate single patients’ data. Horizontal dashed lines indicate upper and lower limits of the normal range (37). Green and blue circles identify patients who were in full or in partial remission, respectively.

Seventy % (17/24) of STEC-HUS sera collected after resolution of STEC infection induced abnormal formation of C5b-9 on activated HMEC-1 (**Figure 7A**), including the 5 patients carrying a rare complement gene variant (**Supplementary Table 1**). The addition of sCR1 to the serum completely normalized C5b-9 formation on HMEC-1.

Notably, the large majority of sera from patients in partial remission caused excessive C5b-9 formation (13 of 15, 87%). Conversely, sera from the majority of patients in full remission yielded normal C5b-9 deposits (**Figure 7A**).

Consistent with the above data, we found that in the overall 24 patients studied after resolution of STEC infection, serum levels of LDH correlated with values of serum-induced C5b-9 formation on ADP-activated cells (Pearson r = 0.47; P = 0.04; n = 20) (**Supplementary Figure 9A**). Also the product of hemoglobin (Hb) and LDH levels (Hb [g/dL] x LDH [IU/L]) correlated with C5b-9 formation on ADP-activated HMEC-1 (Pearson r = 0.55; P = 0.01; n = 20) (**Supplementary Figure 9B**).

To evaluate the impact of serum-induced C5b-9 formation on endothelial dysfunction in STEC-HUS patients after hospital discharge, additional experiments were performed in selected patients with sufficient available serum to evaluate thrombus formation. As shown in **Figure 7B**, a thrombus area above the upper limit of the normal range was observed on ADP-activated HMEC-1 pre-incubated with sera from patients studied in partial remission and causing excessive C5b-9 formation (n = 2). At variance, sera from the patients who were studied in complete remission and showed a normal C5b-9 formation did not exert a prothrombogenic effect on HMEC-1 (n = 2, **Figure 7B**).

Among patients with partial remission and abnormal *ex-vivo* C5b-9 formation, clinical data at a longer follow-up (1–2 years after hospital discharge) were available for four patients (patients #1, #3, #39, and #43). As shown in **Supplementary Table 2**, all these patients remained in partial remission, with 2 of them requiring antihypertensive therapy. A serum sample for the evaluation of *ex-vivo* C5b-9 formation at the longer follow-up was available only for patient #43, and persistently abnormal serum-induced C5b-9 formation on activated HMEC-1 was detected (**Supplementary Table 2**). None of these patients received complement-targeting therapies.

## Discussion

To reproduce the complex interaction between complement and microvascular endothelial cells, which are the primary targets in STEC-HUS, we used *ex-vivo* assays developed in our laboratory, involving the incubation of patient serum on cultured human endothelial cells (32, 38, 39). Our *ex-vivo* results with serum from patients studied during the acute phase of STEC-HUS showed: 1) complement activation on endothelium leading to C3 deposition and formation of C5b-9; 2) endothelial cell shift to a pro-thrombogenic phenotype with upregulated cell surface expression of P-selectin and vWF resulting in, 3) massive formation of platelet thrombi after perfusion with whole blood. All these abnormalities were corrected by iptacopan, a specific inhibitor of the complement alternative pathway.

A large body of evidence in literature documented reduced serum C3 levels and increased plasma levels of complement breakdown products during the acute phase of STEC-HUS, and suggested a functional link between systemic complement activation and microvascular thrombosis (15, 16, 44–46). We describe here that during the acute phase of STEC-HUS, serum-induced C5b-9 formation on endothelial cells correlated with markers of disease activity and of renal dysfunction, whereas circulating C3 and sC5b-9 levels did not, indicating that complement activation on endothelium plays a more relevant role in driving disease manifestations than complement activation in fluid-phase.

We exclude the possibility that abnormal results of the *ex-vivo* test in acute STEC-HUS were the consequence of kidney dysfunction, based on previous studies showing serum-induced C5b-9 deposits in the normal range in patients with chronic kidney disease or ESKD (38, 47).

The complement system can be activated through three pathways: the classical (CP), the lectin (LP), and the alternative pathway (AP), all converging on the cleavage of C3 into C3a and C3b and the formation of the C5 convertase that catalyzes the cleavage of C5, generating the terminal complement products C5a and the C5b-9 lytic complex (48, 49). A major finding of this study is that, while inhibition of the CP and LP pathways only minimally reduced STEC-HUS serum-induced C3 and C5b-9 deposits on endothelial cells, the factor B inhibitor iptacopan effectively blocked C3 and C5b-9 deposition. This indicates that the AP is the primary driver of complement activation at endothelial cell level in acute STEC-HUS.

These findings are in line with previous studies showing that Stxs induce profound alterations in endothelial cells, including direct activation of the complement AP (11, 12). Pre-exposure of microvascular endothelial cells to Stx1 resulted in increased C3 deposition on cell surface upon perfusion with whole blood (12). In addition, Stx2 has been shown to interact with factor H, the major inhibitor of the AP, impairing factor H activity on cell surface, while factor H-mediated fluid phase regulation is maintained (50). Finally, exposure of human glomerular endothelial cells to Stx2 lowered the expression of the surface regulator CD59, which inhibits the formation of C5b-9 complexes (51).

Lipopolysaccharide (LPS), which is released in the gut by STEC, may also enter the systemic circulation and promote activation of the AP (52). In mouse models, LPS has been shown to synergize with Stx in complement activation and HUS-like manifestations (12, 53–57).

One of the most relevant outputs from this work is the evidence that circulating factors during acute STEC-HUS cause a pro-thrombogenic effect on microvascular endothelium. This was documented by massive thrombi on endothelial cells pre-exposed to patient serum and then perfused with whole blood under shear stress comparable to that recorded in the microcirculation. Our findings are fully consistent with clinical evidence showing that organ dysfunction in STEC-HUS results from thrombi that obstruct the microvasculature of various organs, particularly the kidneys, leading to ischemic injury. The key role of complement AP in mediating the above pro-thrombogenic actions in STEC-HUS was demonstrated by results showing that platelet aggregation was prevented by the factor B inhibitor iptacopan added to patients’ sera. These results align with previous studies in a murine model of STEC-HUS, where complement factor B (CFB)-deficient mice were protected against thrombocytopenia, glomerular fibrinogen deposits, and renal dysfunction, compared to wild-type mice (12).

The finding that exposure to STEC-HUS serum led to increased endothelial expression of P-selectin and vWF, and that blocking Weibel-Palade bodies (WPBs) exocytosis fully prevented platelet thrombi, indicates that WPB exocytosis may have a role in AP-mediated prothrombogenic effect on endothelial cells, and may open new therapeutic perspectives.

Interestingly the majority of sera from patients studied after hospital discharge who did not achieve full remission - despite being free of infection - induced abnormal C5b-9 deposition on endothelial cells, suggesting persistent complement activation at the endothelial level even after the acute episode. It is plausible that circulating factors, such as heme from residual hemolysis or substances released from injured cells/tissues, contributed to the elevated *ex-vivo* formation of C5b-9 on endothelial cells (58–60). Supporting this hypothesis, we observed a positive correlation between serum-induced C5b-9 formation and levels of LDH, or the product of hemoglobin and LDH, in patients studied post-resolution of STEC infection. This aligns with the concept that STEC-HUS is a complex disease that may not fully resolve after recovery from the acute phase. Retrospective studies have documented that many STEC-HUS cases result in long-term sequelae of varying severity (6–9), with approximately 30% of patients developing hypertension, impaired renal function, or neurological symptoms within five years post-infection (10).

We speculate that our *ex-vivo* assay of serum-induced C5b-9 formation could serve as an additional valuable tool for long-term monitoring of STEC-HUS patients during follow-up. This approach may help identify patients with persistent status of endothelial-restricted complement activation, facilitating early detection and management of potential sequelae, and ultimately providing an opportunity for improved patient care.

The prognosis of STEC-HUS has improved over time, with 70% of the patients fully recovering thanks to supportive care. However, the fatality rate is still around 1-5% (5) and there is still no specific treatment for the disease. While anti-C5 therapies, such as eculizumab and ravulizumab, have revolutionized the management of aHUS, their efficacy and safety in STEC-HUS is still debated. Reports from small pediatric cohorts with severe STEC-HUS showed significant neurological improvement and hematological normalization with eculizumab (23, 61–63). However, larger studies have failed to demonstrate a clear advantage of eculizumab combined with plasma exchange (PEX) over PEX alone (64, 65). In a retrospective study of 2020, no significant differences in blood pressure, proteinuria, or renal function were observed between eculizumab-treated and untreated patients at 1 and 12-months follow-up (66). More recently, a large phase 3 trial involving 100 pediatric patients with STEC-HUS reported that eculizumab treatment did not improve renal outcome during the acute phase but was associated with reduced long-term kidney sequelae (25).

The limited *in vivo* efficacy of terminal complement pathway inhibitors suggests that additional mechanisms of tissue injury - both complement-dependent distinct from terminal pathway activation and complement-independent - contribute to disease pathogenesis. With regard to complement-mediated mechanisms, Morigi et al. demonstrated that blockade of C3a binding to its receptor (C3aR) reduced platelet aggregation and fibrinogen deposition in the kidneys of Stx/LPS-treated mice, while improving platelet counts and renal function (12). These findings highlight a critical role for early complement activation in the pathophysiology of STEC-HUS, and suggest that therapeutic strategies targeting complement activation upstream of the terminal pathway may be more effective by preventing the generation of both C3- and C5-derived effector molecules. However, complete and non-selective inhibition of C3 activation would compromise essential complement functions required for host defense and STEC clearance. Notably, Morigi et al. also showed that hematologic and renal abnormalities in their model are predominantly driven by alternative pathway activation (12), in line with our *ex-vivo* findings demonstrating the efficacy of iptacopan in inhibiting complement activation and endothelial thrombotic responses in patients with acute STEC-HUS.

Beyond acting as a trigger of complement activation, Stx directly promotes a pro-thrombotic endothelial phenotype through multiple mechanisms, including direct cytotoxicity, disruption of the hemostatic balance, and increased release of pro-inflammatory chemokines (13, 67, 68). Stx also induces up-regulation of tissue factor expression (69), enhances the release of von Willebrand factor (vWF) and ultra-large vWF multimers (70–72), and promotes platelet activation (73). These pathogenic effects may be further amplified over time by immune evasion and persistence of Stx packaged within blood cell-derived microvesicles, which facilitate toxin delivery to target organs (74, 75).

In conclusion, the results of the present study, despite the limitations posed by the relatively small sample size, may provide new therapeutic perspectives for STEC-HUS, a disease that still lacks a specific treatment. Selective blockade of the AP C3 convertase may offer an advantage over anti-C5 or anti-C3 therapies by preserving the function of CP and LP, thereby maintaining critical immune defenses while effectively correcting complement dysregulation in STEC-HUS. This is particularly timely, given that selective AP inhibitors have already been approved or are currently being tested in clinical trials for other rare complement-related diseases, including C3 glomerulopathy and aHUS (76–80).

## Data Availability

The raw data supporting the conclusions of this article will be made available by the authors, without undue reservation.
