# Supplementary material for: Molecular determinants of STEC-HUS: from complement activation to microvascular thrombosis
Source: Front Immunol. 2026 Mar 13;17:1749811. doi: 10.3389/fimmu.2026.1749811 (PMC13021478; doi:10.3389/fimmu.2026.1749811)
Supplement: Supplementary file 1 [file DataSheet1.pdf]

Supplementary Material

Molecular determinants of STEC-HUS:

from complement activation to microvascular thrombosis

Donata Santarsiero[1], Miriam Galbusera[1], Sara Gastoldi[1], Elena Bresin[1], Rossella Piras[1], Marta Alberti[1], Marina Vivarelli[2], Silvia Prandini[1], Sara Conti[1], Federica Zotta[2], Anna Schubart[3], Ariela Benigni[1], Giuseppe Remuzzi[1], Marina Noris[1] and Sistiana Aiello[1]

[1] Istituto di Ricerche Farmacologiche Mario Negri IRCCS, Clinical Research Center for Rare Diseases Aldo e Cele Daccò and Centro Anna Maria Astori, Science and Technology Park Kilometro Rosso, Bergamo, Italy

[2] Division of Nephrology and Dialysis, Bambino Gesù Children’s Hospital IRCCS, Piazza S. Onofrio 4, 00165, Rome, Italy

[3] Department of Immunology, Novartis BioMedical Research, Basel, Switzerland

Supplementary Material

- Supplementary Table S1
- Supplementary Table S2
- Supplementary Figures S1-S9

Supplementary Table S1. Genetic characteristics of STEC-HUS patients.

Patient No.	Rare gene variants	CADD	gnomAD frequency	Classification
**Patient 1**	no	-	-	-
**Patient 2**	no	-	-	-
**Patient 3**	no	-	-	-
**Patient 5**	no	-	-	-
**Patient 6**	CFH (p.S608S)	13.12	0.00008	Likely benign°
**Patient 7**	C3 (p.P1223S)	15.69	0.00001	VUS°
**Patient 8**	no	-	-	-
Patient 9	no	-	-	-
**Patient 11**	no	-	-	-
**Patient 12**	no	-	-	-
Patient 13	del R3 – dupl R4	-	-	Rare structural variant
Patient 14	no	-	-	-
Patient 15	no	-	-	-
Patient 16	no	-	-	-
Patient 17	no	-	-	-
Patient 19	no	-	-	-
Patient 20	no	-	-	-
Patient 21	no	-	-	-
Patient 22	no	-	-	-
Patient 24	no	-	-	-
Patient 26	no	-	-	-
Patient 27	no	-	-	-
Patient 28	no	-	-	-
Patient 29	no	-	-	-
Patient 30	no	-	-	-
Patient 31	no	-	-	-
Patient 32	no	-	-	-
Patient 34	no	-	-	-
Patient 35	no	-	-	-
Patient 36	no	-	-	-
Patient 37	no	-	-	-
**Patient 38**	no	-	-	-
**Patient 39**	no	-	-	-
**Patient 40**	del R1/R3 homozygous	-	-	-
**Patient 41**	C3 (p.M1604T)	13.06	0.00002	VUS°
**Patient 42**	C3 (p.S1608S)	7.17	0.0002	VUS°
**Patient 43**	no	-	-	-
**Patient 44**	no	-	-	-
**Patient 46**	no	-	-	-
**Patient 48**	no	-	-	-
**Patient 49**	no	-	-	-

VUS: variant of uncertain significance.
°ClinVar: https://www.ncbi.nlm.nih.gov/clinvar/
Bold: patients studied at hospital discharge

Supplementary Table S2. Clinical parameters of STEC-HUS patients at longer follow-up (1–2 years after the first follow-up).

Patient No.	Age at follow-up (years)	*Ex vivo* C5b-9 formation (% of NHS)	Clinical parameters[a]					Notes
			Platelets, x10³/µL	Hemoglobin, g/dL	LDH, IU/L	Hp, mg/dL	sCr, mg/dL	
		ADP-activated	Ref range 150-400	Ref range[b]	Ref range 266-500	Ref range 49-246	Ref range[c]	
**Patient 3**	4	nd	245	13.5	535	44	0.47	
Patient 39	3	nd	395	12.9	379	1.72	0.40	Hypertension, controlled with ACE inhibitor (Triatec)
**Patient 1**	4	nd	303	12.8	548	128	0.60	
Patient 43	2	251	461	15.3	743	26	0.26	Hypertension, controlled with ACE inhibitor (Triatec)

Hp, haptoglobin; LDH, lactate dehydrogenase; sCr, serum creatinine; CKD, chronic kidney disease; nd, not determined.
In red: clinical parameters that are outside the age-specific normal range.

[a]Clinical data in the table are those recorded the same days that *ex vivo* C5b-9 formation was evaluated.

[b]Normal range: 10.8-12.5 g/dL for children aged 5 months to 1 year; 11.5-13.5 g/dL for children 1-5 years; 12-14.5 g/dL for children aged 5 to 10 years; 12-15 g/dL for adult female; 13-16 g/dL for adult male.

[c]Normal range: 0.3-0.5 mg/dL for children <1-5 years; 0.5-0.8 mg/dL for children aged 5 to 10 years; 0.5-1.2 mg/dL for children >10 years and adults.

Bold: patients studied also during the acute phase.

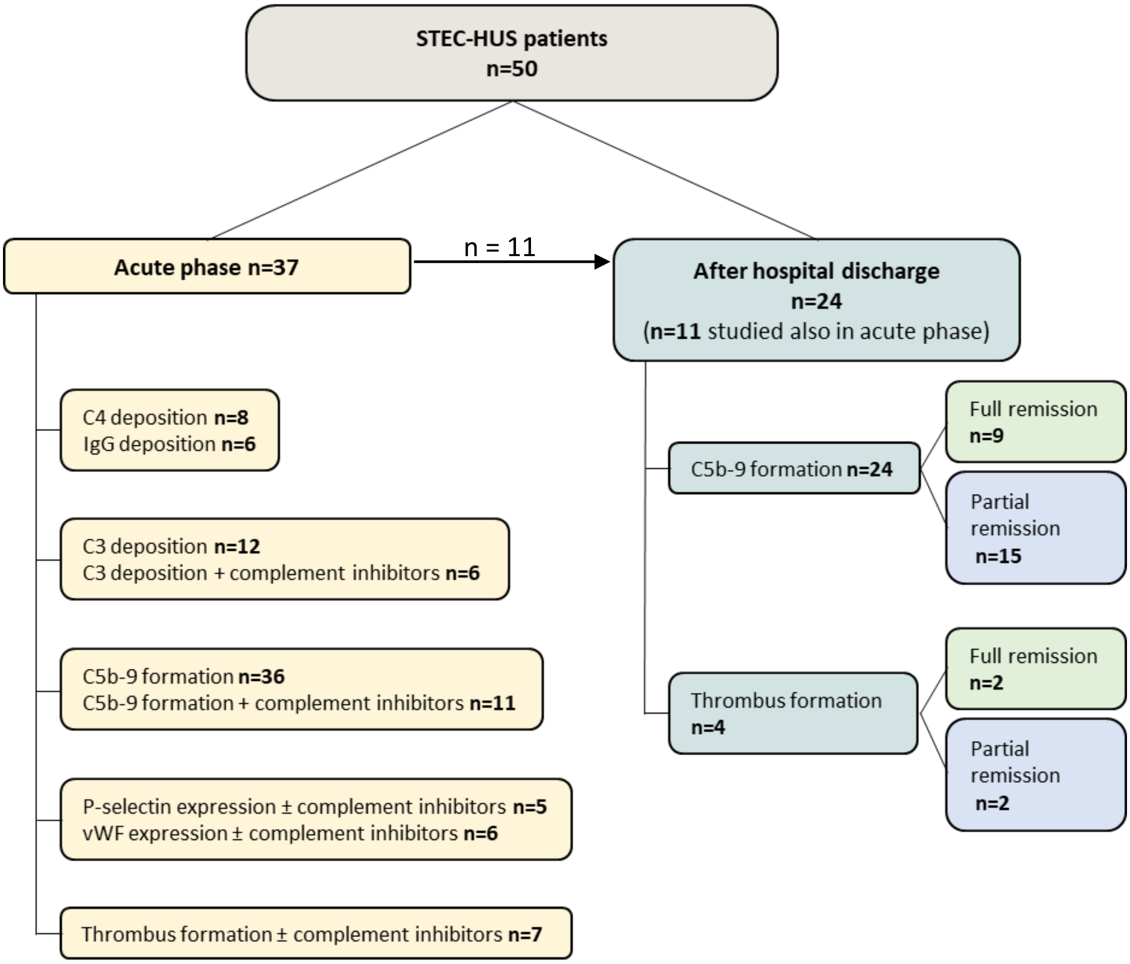

Figure S1. Flow chart summarizing the number of STEC-HUS patients analyzed at each step of the study.

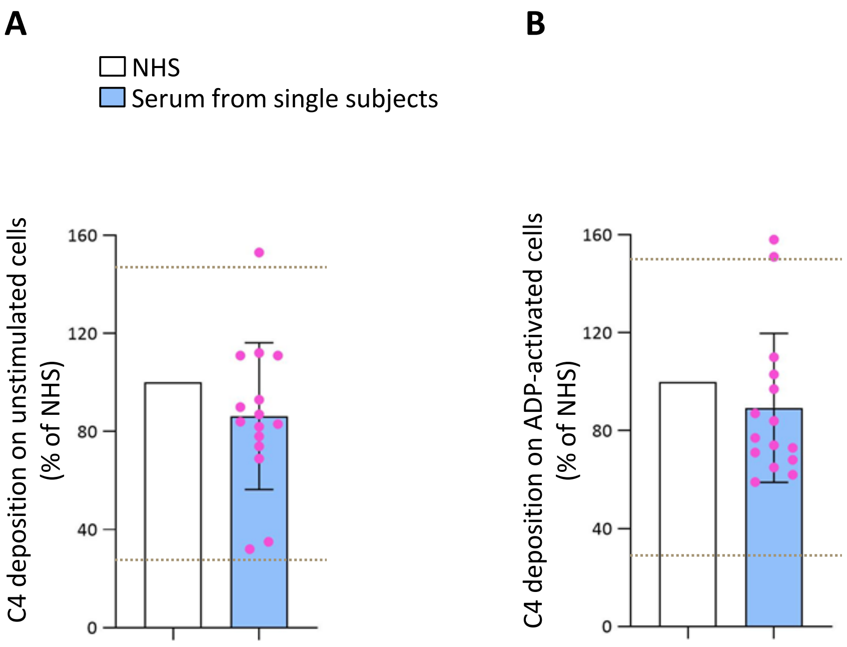

Figure S2. Normal range of C4 deposition.

C4 deposition was evaluated on unstimulated (**A**) and ADP-activated (**B**) HMEC-1, exposed for 4 h to control serum from single healthy subjects (n=15). Results are shown as percentage of stained surface area after incubation with serum from single subjects vs a pool of control sera from 10 healthy subjects (normal human serum, NHS, 100%) run in parallel (mean ± SD). The circles represent single percentages values obtained with each serum. The two dashed lines are the upper and lower limits of the normal range of percentages given by the mean ± 2SD of values obtained with sera from single subjects (normal range was found to be equal to 26-146% on unstimulated cells and 28-150% on ADP-activated cells).

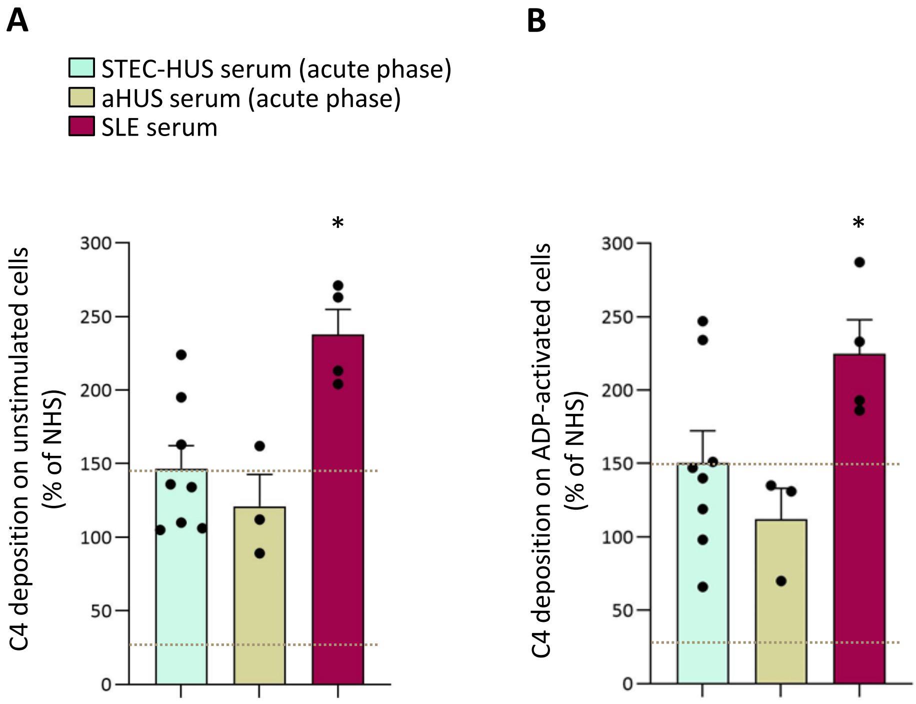

Figure S3. Ex vivo serum-induced C4 deposits on unstimulated and ADP-activated microvascular endothelial cells (HMEC-1) in patients with acute STEC-HUS.
C4 deposition after incubation of unstimulated (**A**) and ADP-activated (**B**) HMEC-1 with serum from patients with acute STEC-HUS, patients with acute aHUS or patients with SLE. Data are expressed as mean ± SD of percentages of serum-induced C4 deposition in respect to a pool of control sera (normal human serum, NHS), run in parallel in each experiment and set as 100%. Circles indicate single patients' data. Horizontal dashed lines indicate upper and lower limits of the normal range (mean ± 2SD). * $P < 0.05$ vs STEC-HUS and aHUS (ANOVA, followed by Tukey's multiple comparisons).

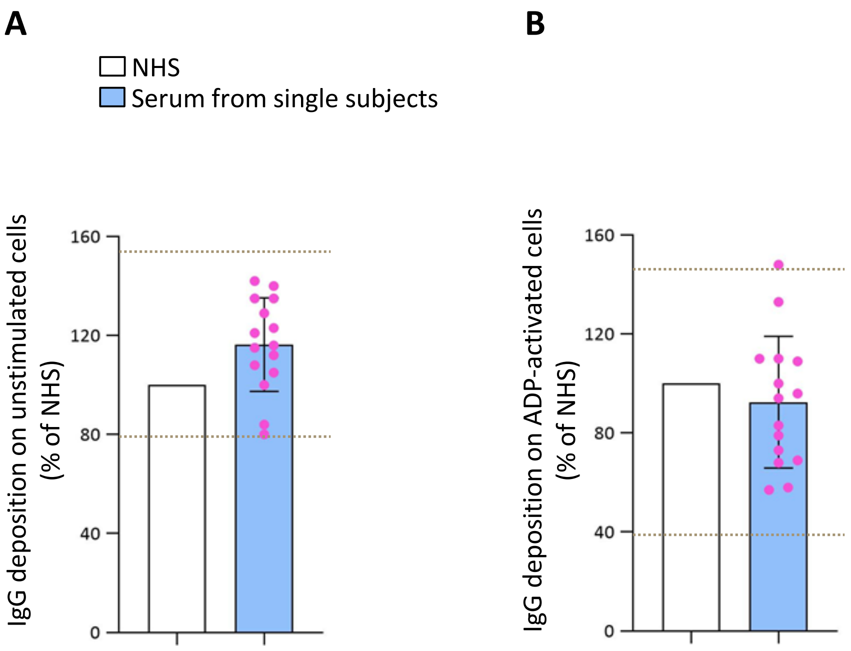

Figure S4. Normal range of IgG deposition.

IgG deposition was evaluated on unstimulated (**A**) and ADP-activated (**B**) HMEC-1, exposed for 4 h to serum from single healthy subjects (serum from single subjects, n=15). Results are shown as percentage of stained surface area after incubation with serum from single subjects vs a pool of control sera from 10 healthy subjects (normal human serum, NHS, 100%) run in parallel (mean ± SD). The circles represent single percentages values obtained with each serum. The two dashed lines indicate the upper and lower limits of the normal range of percentages given by the mean ± 2SD of values obtained with sera from single subjects (normal range was found to be equal to 78-154% on unstimulated cells and 38-146% on ADP-activated cells).

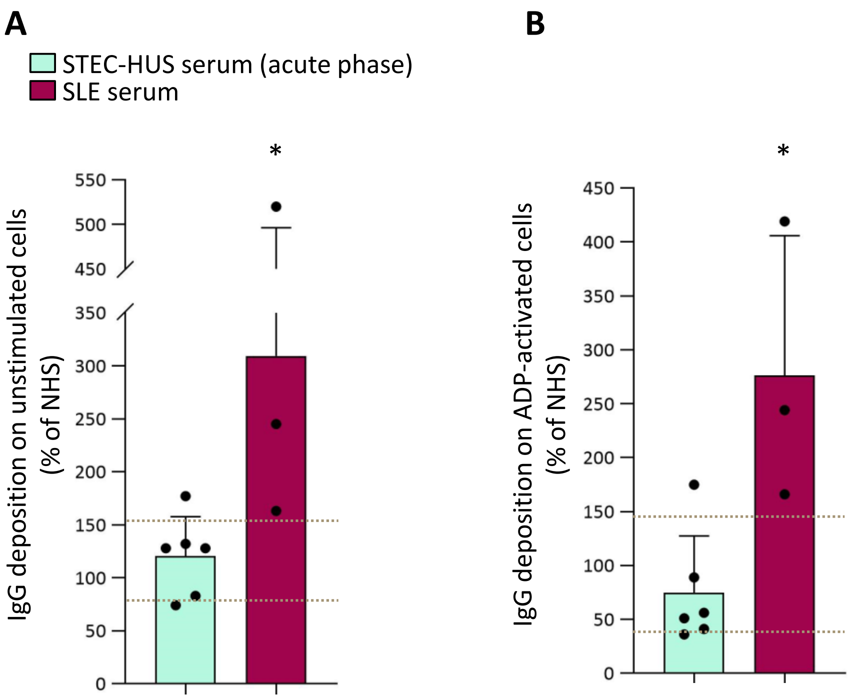

Figure S5. Ex vivo serum-induced IgG deposits on unstimulated and ADP-activated microvascular endothelial cells (HMEC-1) in patients with acute STEC-HUS.

IgG deposition after incubation of unstimulated (**A**) and ADP-activated (**B**) HMEC-1 with serum from patients with acute STEC-HUS or patients with SLE. Data are expressed as mean ± SD of percentages of serum-induced IgG deposition in respect to a pool of control sera (normal human serum, NHS), run in parallel in each experiment and set as 100%. Circles indicate single patients' data. Horizontal dashed lines indicate upper and lower limits of the normal range (mean ± 2SD). * $P < 0.05$ vs STEC-HUS (Student's t test for unpaired samples (A), Mann-Whitney test (B)).

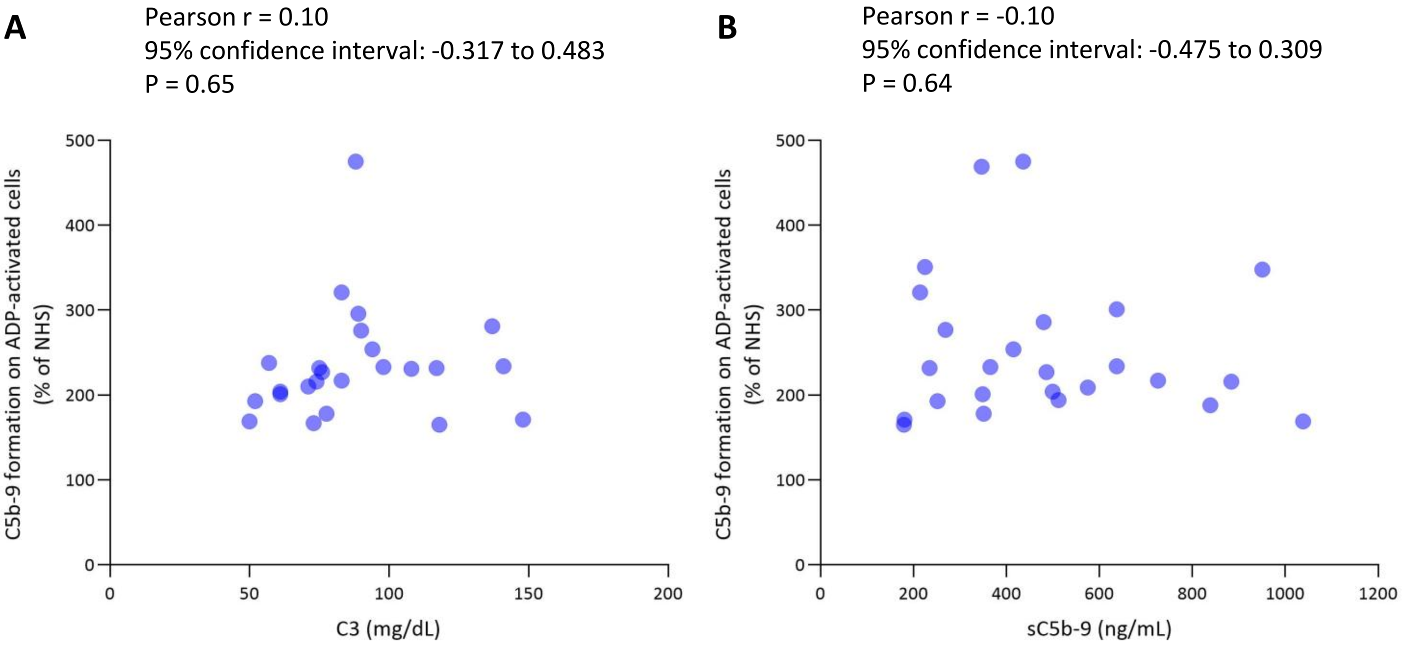

Figure S6. Ex-vivo serum-induced C5b-9 formation on ADP-activated endothelial cells does not correlate with either serum levels of C3 or plasmatic sC5b-9 levels in acute STEC-HUS.

Ex-vivo serum-induced C5b-9 formation (% of NHS) on ADP-activated HMEC-1 plotted against serum C3 levels (mg/dL) **(A)** and plasmatic sC5b-9 levels (ng/mL) **(B)**.

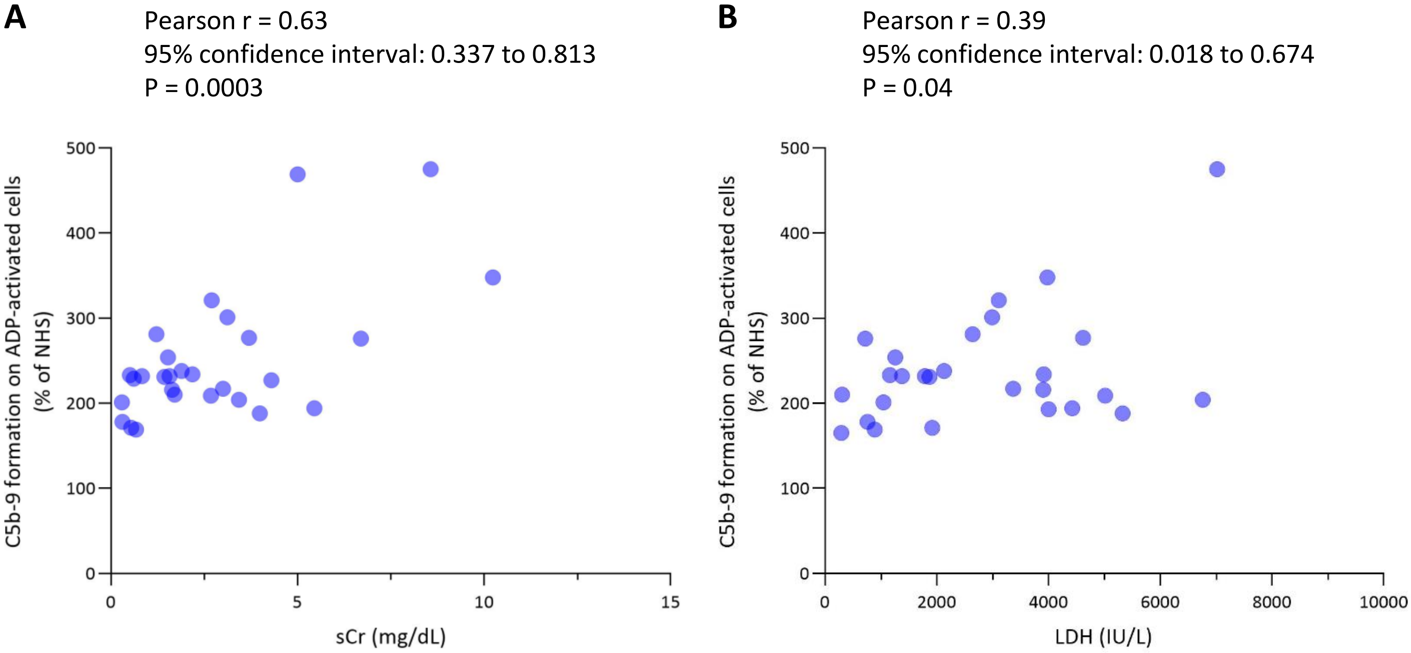

Figure S7. Ex-vivo serum-induced C5b-9 formation on ADP-activated endothelial cells correlates with serum creatinine and with LDH levels in acute STEC-HUS.

Ex-vivo serum-induced C5b-9 formation (% of NHS) on ADP-activated HMEC-1 plotted against serum creatinine levels (mg/dL) (**A**) and LDH levels (IU/L) (**B**).

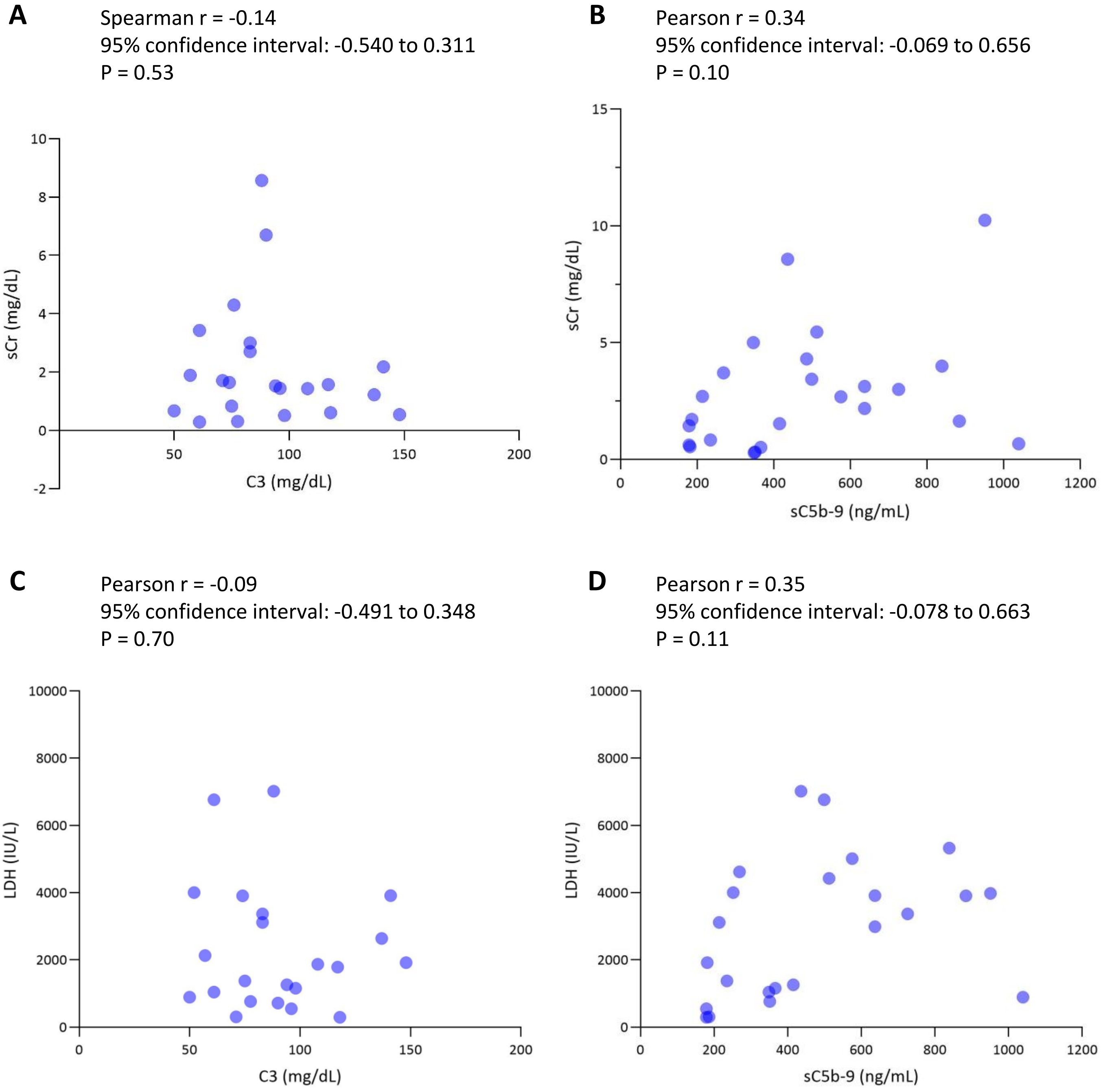

Figure S8. Serum creatinine levels and LDH values do not correlate with either serum levels of C3 or plasmatic sC5b-9 values in acute STEC-HUS.

Serum creatinine levels (mg/dL) plotted against C3 serum levels (mg/dL) **(A)** and against plasmatic sC5b-9 levels (ng/mL) **(B)**. LDH levels (IU/L) plotted against C3 serum levels (mg/dL) **(C)** and against plasmatic sC5b-9 levels (ng/mL) **(D)**.

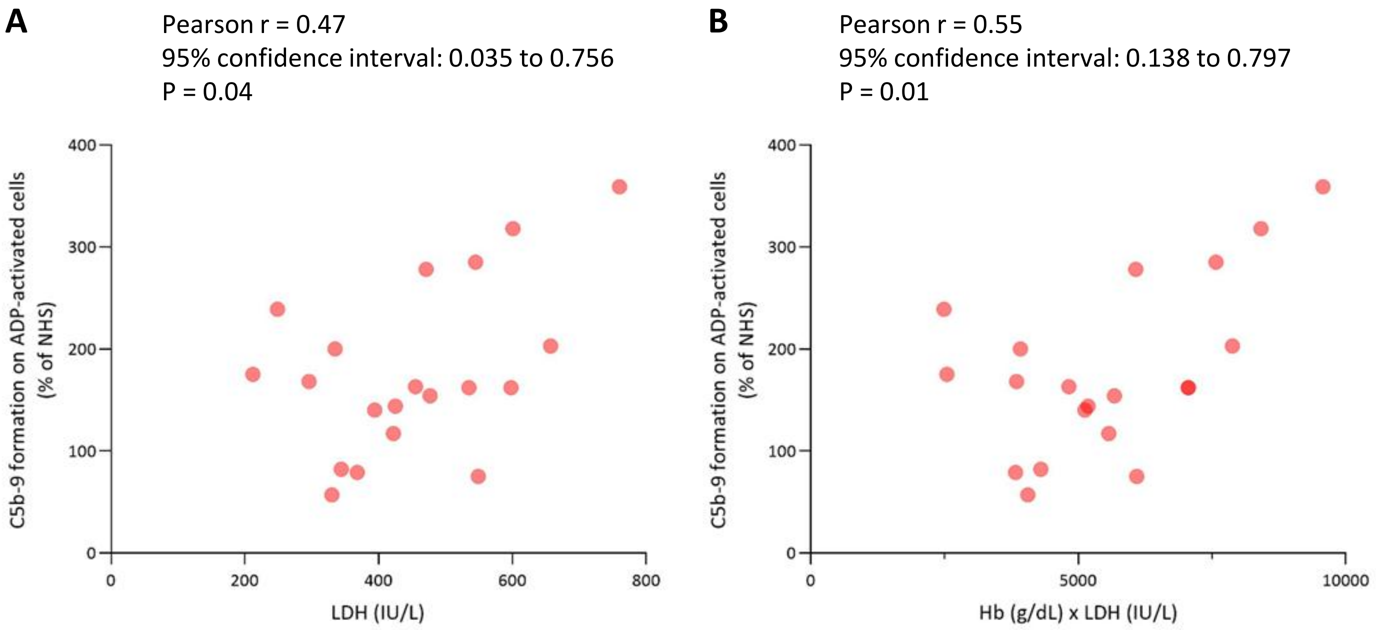

Figure S9. Ex-vivo serum-induced C5b-9 formation on ADP-activated endothelial cells correlates with LDH levels and with the product Hb x LDH in STEC-HUS patients out of STEC infection.

Ex-vivo C5b-9 formation (% of NHS) induced by serum from patients out of STEC infection on ADP-activated HMEC-1 plotted against LDH levels (IU/L) (**A**) and against the product of haemoglobin and LDH levels (Hb (g/dL) x LDH (IU/L)) (**B**).
